# Adaptation to ER Stress Is Mediated by Differential Stabilities of Pro-Survival and Pro-Apoptotic mRNAs and Proteins 

**DOI:** 10.1371/journal.pbio.0040374

**Published:** 2006-11-07

**Authors:** D. Thomas Rutkowski, Stacey M Arnold, Corey N Miller, Jun Wu, Jack Li, Kathryn M Gunnison, Kazutoshi Mori, Amir A. Sadighi Akha, David Raden, Randal J Kaufman

**Affiliations:** 1 Howard Hughes Medical Institute, University of Michigan Medical Center, Ann Arbor, Michigan, United States of America; 2 Department of Biological Chemistry, University of Michigan Medical Center, Ann Arbor, Michigan, United States of America; 3 Department of Biophysics, Graduate School of Science, Kyoto University, Kyoto, Japan; 4 Department of Pathology, University of Michigan Medical Center, Ann Arbor, Michigan, United States of America; 5 Department of Chemical Engineering, University of Delaware, Newark, Delaware, United States of America; 6 Department of Internal Medicine, University of Michigan Medical Center, Ann Arbor, Michigan, United States of America; University of California San Francisco, United States of America

## Abstract

The accumulation of unfolded proteins in the endoplasmic reticulum (ER) activates a signaling cascade known as the unfolded protein response (UPR). Although activation of the UPR is well described, there is little sense of how the response, which initiates both apoptotic and adaptive pathways, can selectively allow for adaptation. Here we describe the reconstitution of an adaptive ER stress response in a cell culture system. Monitoring the activation and maintenance of representative UPR gene expression pathways that facilitate either adaptation or apoptosis, we demonstrate that mild ER stress activates all UPR sensors. However, survival is favored during mild stress as a consequence of the intrinsic instabilities of mRNAs and proteins that promote apoptosis compared to those that facilitate protein folding and adaptation. As a consequence, the expression of apoptotic proteins is short-lived as cells adapt to stress. We provide evidence that the selective persistence of ER chaperone expression is also applicable to at least one instance of genetic ER stress. This work provides new insight into how a stress response pathway can be structured to allow cells to avert death as they adapt. It underscores the contribution of posttranscriptional and posttranslational mechanisms in influencing this outcome.

## Abstract 2

Cells can adapt to chronic, mild ER stress without undergoing apoptosis. This is in part because the expression of pro-apoptotic pathway components is short-lived, which allows adaptive pathways to predominate.

## Introduction

The ability to sense and respond to the accumulation of misfolded proteins is a central component of the cellular defense against environmental insult. The importance of protein folding in maintaining cellular function is underscored by the observation that, for every compartment in which proteins fold, both a quality control apparatus and a system for sensing perturbation within that compartment exist to shepherd the folding process [1].

The accumulation of unfolded or misfolded proteins in the endoplasmic reticulum (ER) lumen results in activation of the unfolded protein response (UPR). Protein folding stress on the ER can result from a number of insults, including exposure to pharmacological agents that perturb protein folding, genetic mutation of ER chaperones or chaperone substrates, viral infection, metabolic demands, and even normal differentiation and function of professional secretory cells [2]. The mammalian cell senses the accumulation of unfolded proteins that these insults generate through the action of three key ER-resident transmembrane proteins—PERK, IRE1, and ATF6 [3]. Although the exact mechanisms are still not well described, each of these three proximal sensors of ER stress is thought to be activated in part by titration of the ER chaperone BiP away from interactions with these proteins in favor of binding to misfolded proteins in the lumen [4–7]. Dissociation from BiP frees the serine/threonine kinases PERK and IRE1 for homodimerization, autophosphorylation, and activation [8,9], and also liberates ATF6 for transit to the Golgi, where it is cleaved by S1P and S2P processing enzymes to yield a cytosolic transcription factor that transits to the nucleus and activates gene expression [6,10–14].

PERK is a member of a family of protein kinases that phosphorylate the alpha subunit of the cytosolic eukaryotic translation initiation factor eIF2, resulting in an inhibition of *80S* ribosome assembly and protein synthesis. As this inhibition is a relatively rapid consequence of UPR activation [15,16], one of the first manifestations of the ER stress response is a transient decrease in the load of proteins entering the ER [17]. One consequence of eIF2α phosphorylation is the selective translation of certain mRNAs that require inefficient ribosome assembly for proper *AUG* codon recognition. The best characterized of these is the transcription factor ATF4 [18–20] that activates the transcription of genes involved in, among other pathways, amino acid biosynthesis and redox balance—genes that are important in the response to stresses in addition to ER stress. Therefore, a long-term consequence of PERK activation is up-regulation of a transcriptional program that helps the cell offset the pleiotropic consequences of protein misfolding in the ER [20–22].

The lumenal domains of IRE1 (for which there is a ubiquitously expressed alpha form and a beta form restricted to the intestinal epithelium [23–25]) and PERK are homologous and functionally interchangeable [4,5], supporting a common mechanism of activation. However, IRE1 phosphorylation activates an endoribonuclease activity in the cytosolic domain of the protein, leading to the catalytic removal of a 26-base intron from the mRNA of a single gene, *Xbp1* [26–29]. This splicing and religation event results in a translational frameshift to produce an active XBP1 transcription factor. The subset of genes dependent upon XBP1 action remains only partially characterized, but in general seems to encompass those involved in facilitating protein degradation [30,31]. Although XBP1 splicing is, like eIF2α phosphorylation, a relatively rapid consequence of UPR activation, the effects of IRE1 activation on gene expression manifest somewhat later in the response, probably because basal expression of *Xbp1* mRNA is low, and is itself up-regulated by UPR activation [27,29].

ATF6 is a member of an emerging family of transmembrane proteins cleaved by S1P and S2P to liberate active cytosolic transcription factors in response to ER stress. ATF6α and -β are ubiquitously expressed [32], whereas expression of other related proteins, such as CREBH, OASIS, luman, and TISP40, is restricted to particular tissues [33–36]. Overexpression of the active form of ATF6α results in transcriptional activation of genes encoding ER chaperones, such as BiP, GRP94, PDI, and calreticulin [21,37].

A paradox of the UPR is that the response leads to the simultaneous activation of both adaptive and pro-apoptotic pathways. The best characterized of these pro-apoptotic pathways is production of the CHOP/GADD153 transcription factor, which is regulated by ATF4 and possibly ATF6 [21,38,39]. Deletion of *Chop* partially protects both cells and animals from ER stress-mediated cell death [40]. The mechanism by which CHOP leads to cell death is not yet known, but it has recently been suggested that CHOP activates transcription of GADD34, which interacts with protein phosphatase I to catalyze eIF2α dephosphorylation [17,41–43]. By thereby promoting the resumption of protein synthesis in a cell already burdened by unfolded proteins in the ER, GADD34 might spur an attendant production of reactive oxygen species [22,44]. It has also been proposed that CHOP leads to transcriptional repression of the anti-apoptotic BCL-2 protein [45], and to up-regulation of the death receptor family member DR5 [46]. The UPR is known to initiate other pro-apoptotic events as well, including JNK phosphorylation (thought to depend on IRE1 activation), relocalization of BCL-2 family members, cleavage of ER-specific caspases, p53 activation, and disruption of cellular calcium homeostasis [47–49]. In general, preventing the initiation of these events singly by genetic ablation confers a degree of protection to cells from ER stress, but it is not understood how the cell is able to escape death when the misfolding problem can be alleviated.

A common viewpoint for rationalizing the simultaneous activation of pro-apoptotic and pro-survival pathways by the UPR is that death is an inevitable consequence of persistent ER stress (e.g., [50,51]), and indeed, prolonged and severe pharmacological perturbation of ER function in cultured cells does indeed lead to death(e.g., [52,53]). However, the documented instances of physiological activation of the UPR argue that there are many circumstances when the cell is able to selectively utilize the pro-survival aspects of the response. For instance, the development and differentiation of professional secretory cells such as B lymphocytes [54] requires that these cells tolerate the increasing burden of secretory protein synthesis and respond not with cell death, but with an increased protein folding and processing capacity. Likewise, many of the pathologies for which UPR activation has been implicated, such as hepatitis [55], diabetes [56], and various neurodegenerative diseases [57], occur over a time course of years. Under these circumstances, even if cell death occurs to a small extent, the majority of cells chronically exposed to such protein folding insults must survive and adapt. However, experimental induction of the UPR has as yet shed little light on how the UPR can facilitate adaptation given its pro-apoptotic components.

Given the simultaneous activation of pro-survival and pro-apoptotic pathways by the UPR, how are cells able to selectively utilize the adaptive program of the UPR? To address this question, we created a tractable experimental system in which UPR activation was dissociated from cell death. We demonstrate that adaptation to stress is an intrinsic consequence of low-level activation of the UPR, and is accompanied by changes in the patterns of protein expression that are qualitatively distinct from the UPR as induced by severe acute stress. We also provide evidence that an adaptive response to chronic stress is a consequence not of selective activation of proximal ER stress sensors, but of preferential stabilities of mRNAs and proteins that facilitate adaptation versus those that lead to cell death. Finally, these findings are supported by at least one instance of genetic, rather than pharmacological, stress, and so are potentially applicable to physiological and pathophysiological induction of the UPR as well.

## Results/Discussion

### Experimental Approach

Because the mechanisms whereby the UPR can become a predominantly adaptive response, rather than an apoptotic one, are not known, we set about reconstituting an adaptive response in a simple and tractable experimental system, with three goals in mind: (1) to characterize how an adaptive UPR differs from a terminal UPR; (2) to identify a mechanism by which the UPR could facilitate adaptation rather than death; and (3) to determine whether such findings could be extended to non-pharmacological, and thus more physiologically salient, examples of ER stress.

To be considered adaptive, the response elicited must prevent the cell population from succumbing to apoptosis, allow for cell proliferation despite UPR activation, and desensitize cells to the stress induced by the perturbant. As a first step toward this goal, we asked whether conditions could be found that allow cultured cells to proliferate despite an activated UPR. We chose as our experimental cell type mouse embryonic fibroblasts (MEFs), because, as primary non-immortalized cells, they are less likely than immortalized cell lines to be intrinsically resistant to stress. We used as stressors thapsigargin (TG), which blocks calcium reuptake into the ER lumen, and thus depletes calcium from the organelle, and tunicamycin (TM), which inhibits N-linked glycosylation in the ER. TG induces both rapid flux of calcium from the ER, and rapid re-establishment of calcium equilibrium in the ER. Thus TG treatment disrupts protein folding in a fairly vigorous and focal manner, and quickly activates the UPR [58]. In contrast, TM relies upon protein biosynthesis to exert its effect and so is a less robust and slower-acting inducer of ER protein misfolding [59]. Therefore, both of these stressors lead to accumulation of unfolded proteins and UPR activation, but by different mechanisms and with different kinetics, and both are relatively specific inducers of ER stress.

### Proliferation Despite ER Perturbation and UPR Activation

**Figure 1:**
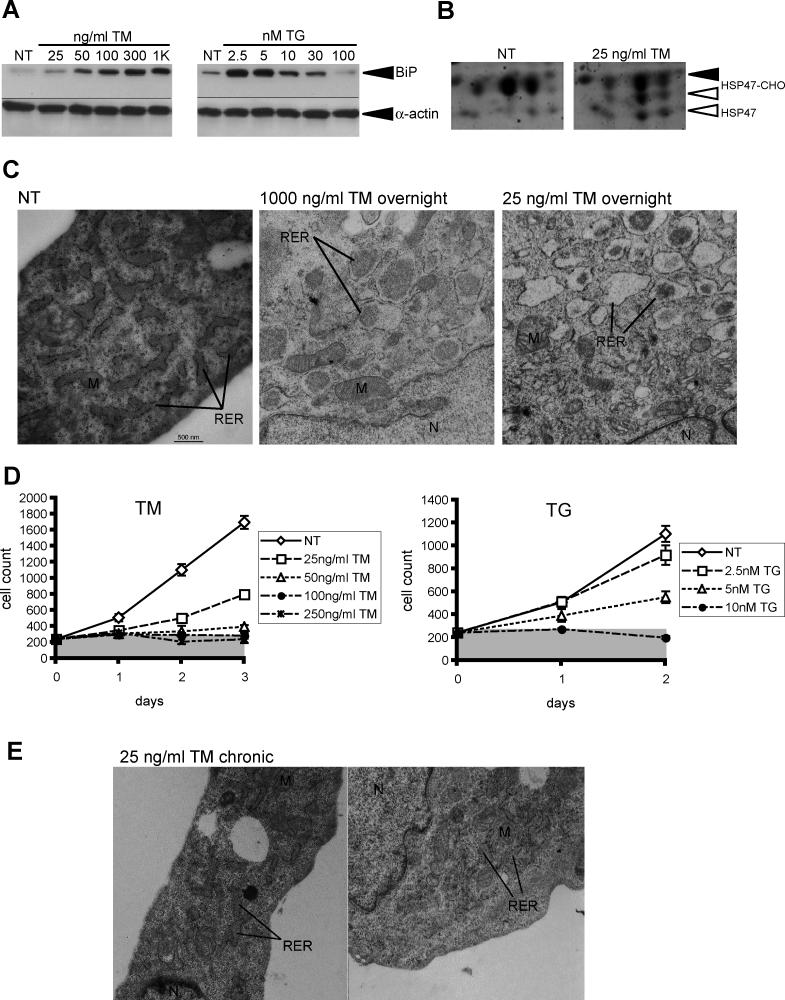
UPR Activation Can Permit Cell Survival and Proliferation (A) MEFs were cultured for 24 h in the presence of increasing concentrations of TM or TG. Cell lysates were then probed by immunoblot with antibodies specific for BiP, or α-actin as a loading control. (B) MEFs were cultured in the presence of 25-ng/ml TM or vehicle. The ER fraction was isolated from each by differential centrifugation, and was then separated by two-dimensional SDS-PAGE. The gels were stained for total protein with SYPRO Ruby. The series of spots corresponding to the ER-resident sentinel glycoprotein HSP47, identified by mass spectrometry, is indicated, with fully glycosylated (HSP47-CHO, filled arrow), underglycosylated (HSP47-CHO, open arrow) and unglycosylated (HSP47, open arrow) species visible. There are four isoforms of HSP47 that differ in isolectric point. Treatment of cells in a higher concentration of TM confirmed that the faster-migrating species are under- or non-glycosylated forms of the protein. (C) MEFs were treated overnight in the indicated concentration of TM. Cells were fixed in 2.5% glutaraldehyde, then prepared for transmission electron microscopic analysis. Nuclei (N), mitochondria (M), and rough ER (RER) are indicated. All images are at 25,000× magnification. Scale bar represents 500 nm. (D) MEFs were plated in replicate and exposed to varying concentrations of TM or TG, with the media and stressor refreshed daily. At each day, cells from individual plates were washed and trypsinized, and the cell concentration in the disrupted cell suspension was determined using an automated cell counter. Each suspension was counted twice, and error bars represent means ± SDM from triplicate plates. The numbers given on the *y*-axis represent cell number per milliliter in the diluted cell suspension. The gray bar is extended from the starting cell count plus the standard deviation, to allow net growth of the culture, or lack thereof, to be more readily assessed. (E) Cells were cultured for 6 d in the presence of 25-ng/ml TM, refreshed daily, and analyzed by electron microscopy for ER structure as in (C).

TM is commonly applied to cells during experimental induction of the UPR in the range of 2–10 μg/ml (2.4–12 μM); likewise, 100–500 nM TG also induces robust UPR activation, and both stressors at these concentrations lead to massive cell death in MEFs (e.g., [52,53]). We found that the concentrations of both agents could be reduced by two orders of magnitude and still elicit an ER stress response, as assessed by increases in the steady-state level of the ER chaperone BiP (Figure 1A), which is the most commonly used marker for UPR activation.

A low concentration of TM (25 ng/ml; 30 nM) produced a modest but distinct effect on protein glycosylation, leading to underglycosylation of the ER-resident chaperone HSP47, but having no discernible effect on the glycosylation of the translocon accessory component TRAPα (Figure 1B and unpublished data). Despite this modest effect, 25-ng/ml TM leads to ER perturbation. Transmission electron microscopic images of untreated MEFs consistently revealed the ER as a series of ribosome-studded finger-like projections distributed throughout the cytoplasm (*n =* 12/13) (Figure 1C and Table S1). Cells treated overnight with a high concentration of TM (1–2 μg/ml) displayed gross distortion of ER morphology, with the organelle appearing in most cells as dilated vesicles containing localized regions of electron-dense material, possibly representing protein aggregates (*n =* 10/14). In other cells, the ER took an apparently fragmented form, with the organelle seen as much smaller vesicles (*n =* 2/14). Surprisingly, a low concentration of TM (25 ng/ml), despite being a much less-severe stressor, produced a similar effect. Many cells (*n =* 6/12) showed dilated ER, with regions of electron-dense material within, whereas other cells showed apparent ER fragmentation or vesiculation (*n =* 3/12). Therefore, the UPR induced by 25-ng/ml TM is likely a direct consequence of protein misfolding and ER perturbation. The effects of TG on ER morphology were more subtle, with a small but significant number (4/14) showing various aberrant ER structures upon exposure to 2.5 nM TG (Figure S1 and Table S1).

Despite eliciting UPR activation and ER perturbation, 25–50-ng/ml TM allowed for net growth of the cell population, albeit at a slower rate than untreated cells (Figure 1D). In fact, cells treated continuously at these concentrations achieved confluence, and could be passaged for as long as their untreated counterparts, eventually achieving a near-normal growth rate despite the continued presence and activity of TM in the culture (Figures S2 and S3). In contrast to cells newly exposed to TM, cells continuously cultured in TM for a longer period of time (~2 wk) showed very little ER perturbation in response to the drug (Figure 1E and Table S1), with most cells (*n =* 11/15) showing normal ER morphology. Annexin V staining revealed very little apoptosis in cells treated with 25-ng/ml TM, suggesting that the attenuation of proliferative capacity in cells treated under such conditions is more likely to be attributable to a growth delay than to selection for small drug-resistant subpopulations (Figure S4). In contrast to their response to low doses of TM, the cell population experienced unrecoverable cell death and net loss of cell number at concentrations of TM at or above 100 ng/ml (120 nM). Likewise, 2.5–5 nM TG allowed for cell proliferation and passage despite the continued responsiveness of these cells to the pharmacological effect of TG (Figure S3), whereas higher concentrations did not (Figure 1D). These results indicate that the UPR can be activated by diverse stressors without necessitating cell death, and suggest that cells become resistant to the perturbing effects of mild disruption of protein folding in the ER.

### Survival Does Not Require Selective Activation of UPR Stress Sensors

**Figure 2:**
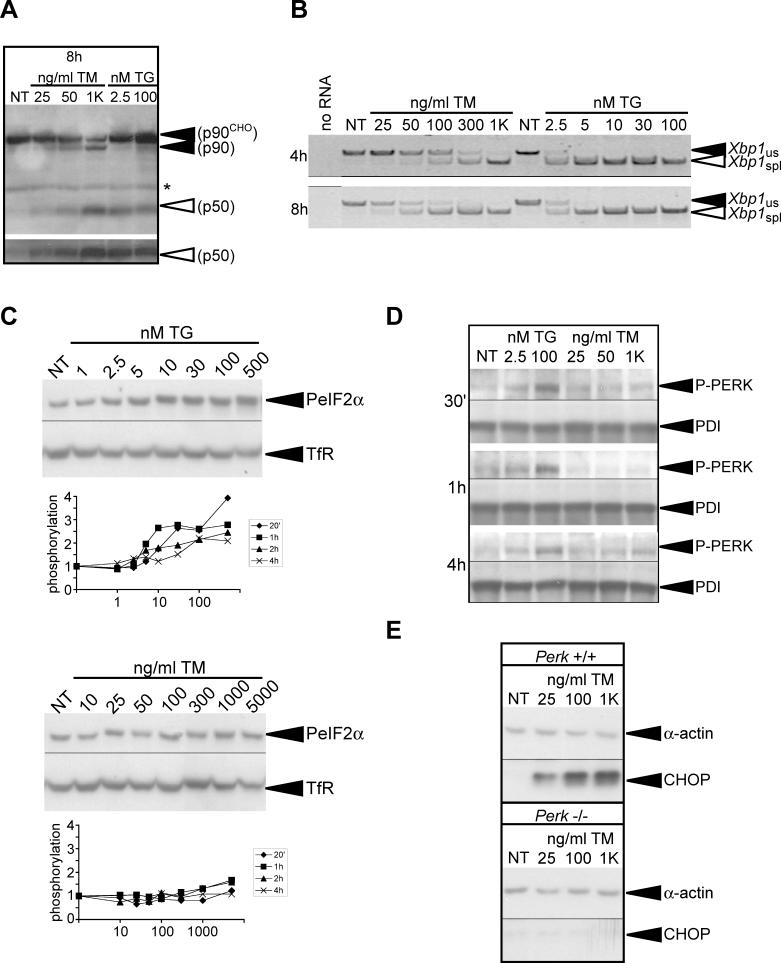
Mild ER Stress Activates ATF6, IRE1, and PERK (A) Following treatment for 8 h in the indicated concentrations of TM or TG, MEFs were probed for expression of ATF6α. The uncleaved forms, both glycosylated (p90^CHO^) and unglycosylated (p90), are visible, as is the active (p50) form. The bottom panel is a darker exposure of just the active form of ATF6α. The asterisk denotes a nonspecific background band. (B) Total RNA was isolated from MEFs treated for 4 or 8 h with the indicated concentrations of TM or TG. RT-PCR with gene-specific primers was used to simultaneously detect both spliced (spl) and unspliced (us) *Xbp1* mRNA. The image is presented in black-and-white inverted form for greater visual clarity. (C) After varying intervals of exposure to TG (top panel) or TM (bottom panel), lysates from MEFs were prepared and probed by immunoblot with antibodies specific for phosphorylated eIF2α or transferrin receptor (TfR) as a loading control. The blot from the 2-h time point is shown as representative. The extent of eIF2α phosphorylation relative to untreated control cells was quantitated by densitometry, and is shown in graphical form for each time point below the blots. (D) Cells were treated with the indicated concentrations of TG or TM for 30 min, 1 h, or 4 h, and protein lysates were harvested for immunoblot with antibodies specific for the phosphorylated form of PERK (P-PERK), or PDI as a loading control. Specificity of the phospho-PERK antibody was confirmed using lysates from *Perk^−/−^* cells (unpublished data). (E) Lysates from *Perk^−/−^* or wild-type matched MEFs were prepared after 24 h of exposure to TM as indicated. Levels of CHOP and α-actin were assessed by immunoblot. Images for *Perk^+/+^* and *Perk^−/−^* cells were taken from the same exposure.

One possible mechanism to explain cell survival during mild ER stress would be selective activation of one or more of the proximal sensors of ER stress, such that pro-apoptotic cascades were not induced. To test this hypothesis, we monitored the activation of ATF6α, IRE1, and PERK in response to varying concentrations of TM and TG. The active, cleaved N-terminal fragment of ATF6α was directly detected by immunoblot during treatment with low concentrations of either TM (25–50 ng/ml) or TG (2.5 nM) (Figure 2A). Similarly, treatment with either 25–50-ng/ml TM or 2.5–5 nM TG resulted in splicing of *Xbp1* mRNA, albeit less robustly than when much higher concentrations of either drug were applied (Figure 2B). PERK activation can be assessed by measuring the phosphorylation of its target, eIF2α, using an antibody reactive only toward the phosphorylated form of eIF2α. By this assay, PERK was activated (~1.4-fold at maximum) at concentrations of TG as low as 2.5 nM, although as with *Xbp1* splicing, this activation was much less robust than at higher doses of TG (Figure 2C). In contrast, TM was a much poorer inducer of eIF2α phosphorylation, which was seen reliably over background levels only at the highest concentrations of the drug (Figure 2C). Similar results were obtained using an antibody that directly detected phosphorylated PERK (Figure 2D). This result was mirrored in translational attenuation, which occurs as a consequence of eIF2α phosphorylation. Both high and low concentrations of TG produced a rapid and robust inhibition of protein synthesis, whereas 1-μg/ml TM had only a relatively modest effect, and 25-ng/ml TM had very little effect (Figure S5). However, we also found that expression of CHOP protein was up-regulated during treatment with 25-ng/ml TM, and this up-regulation was completely abrogated in *Perk*-deficient MEFs (Figure 2E). Therefore, we conclude that PERK is indeed activated by low concentrations of TM, and that PERK can be activated to a small extent even when its activation is not readily detected by the standard measures (eIF2α phosphorylation and inhibition of protein synthesis). Based on both direct and indirect measures of activation, these data support the notion that low concentrations of TM and TG activate PERK, IRE1, and ATF6α, and that *selective* activation of proximal stress signaling molecules is not required for survival.

### Differences in Protein Expression Distinguish Adaptive from Terminal UPR Activation

**Figure 3:**
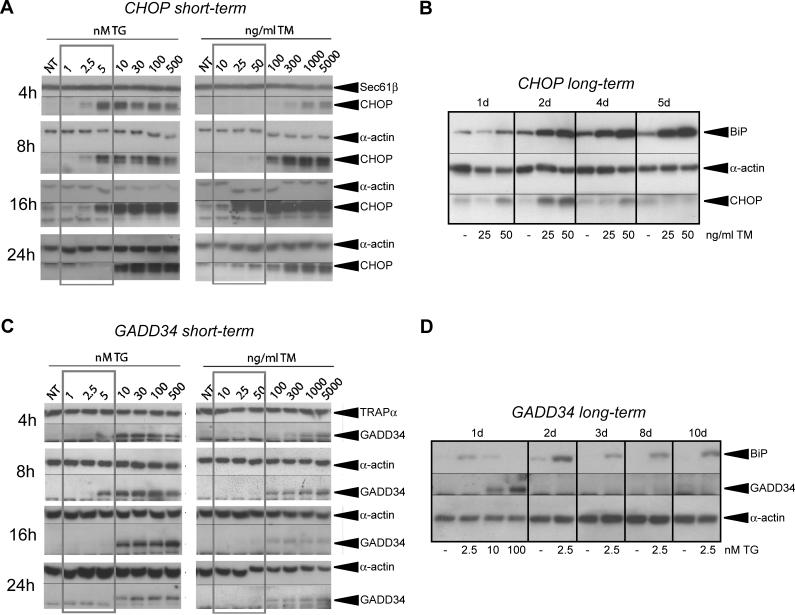
Expression of CHOP and GADD34 Correlates with Cell Fate (A) MEFs were treated with increasing concentrations of TG or TM for the indicated times, followed by cell lysis and immunoblot for CHOP, or α-actin or Sec61β to judge loading. The concentrations outlined by the gray box are those that allow for survival. For every time-point, the TM and TG panels were taken from the same blot, and the same exposure time. (B) MEFs were treated for up to 5 d in the continuous presence of 25- or 50-ng/ml TM, with the media and stressor refreshed each day; and expression of CHOP, BiP, and α-actin was probed by immunoblot. (C) Same as in (A), probing instead for GADD34, or α-actin or TRAPα as loading controls. (D) Cells treated continuously in TG at the indicated concentrations were probed for expression of BiP, GADD34, and α-actin by immunoblot.

Another way in which survival could be favored as an outcome is if an adaptive UPR diverges from a terminal UPR, not at the level of activation, but in the expression of downstream genes. To pursue this idea, we monitored the expression of CHOP protein over a 24-h time course, in response to both lethal and sub-lethal concentrations of TM and TG, asking whether its expression, as representative of a pro-apoptotic pathway, was a predictor of cell death. For TG treatment we found that, although low concentrations of TG that allowed for survival and proliferation resulted in production of CHOP protein, its up-regulation was lost by 24 h of treatment under conditions that cells could survive, but not at higher concentrations that prohibited survival (Figure 3A, left panel). Similarly, low concentrations of TM induced CHOP expression, but this up-regulation peaked after approximately 16 h of treatment, and was diminished by 24 h (Figure 3A). Over a longer time course of TM treatment during a separate experiment, CHOP up-regulation was diminished substantially, despite the media and stressor being refreshed daily, and also despite persistent up-regulation of the UPR-responsive ER chaperones BiP and GRP94 (Figures 3B and S6). For conditions of stress—either TM or TG—that prohibited culture proliferation, CHOP up-regulation was never found to diminish to levels comparable to untreated cells (unpublished data). These data are consistent with the notion that the UPR is activated in its entirety by mild ER stress, but that there is a divergence in the expression of downstream proteins depending upon whether the outcome is survival or apoptosis.

As a transcription factor, CHOP itself is unlikely to be intrinsically apoptotic; it more likely alters the expression of one or more downstream genes that facilitate cell death. Analyzing expression of one likely target of CHOP, GADD34 protein, we found that its production correlated even more strongly with cell fate. Its production was readily and consistently detected upon lethal exposure to TM or TG, but at lower doses its production was, at most, transient (Figure 3C). Moreover, the absence of GADD34 production occurred despite persistent BiP up-regulation (Figure 3D). These data suggest that CHOP expression in an adaptive response is not robust enough to effect changes in the expression of downstream genes.

### Attenuation of UPR Signaling Accompanies Adaptation

**Figure 4:**
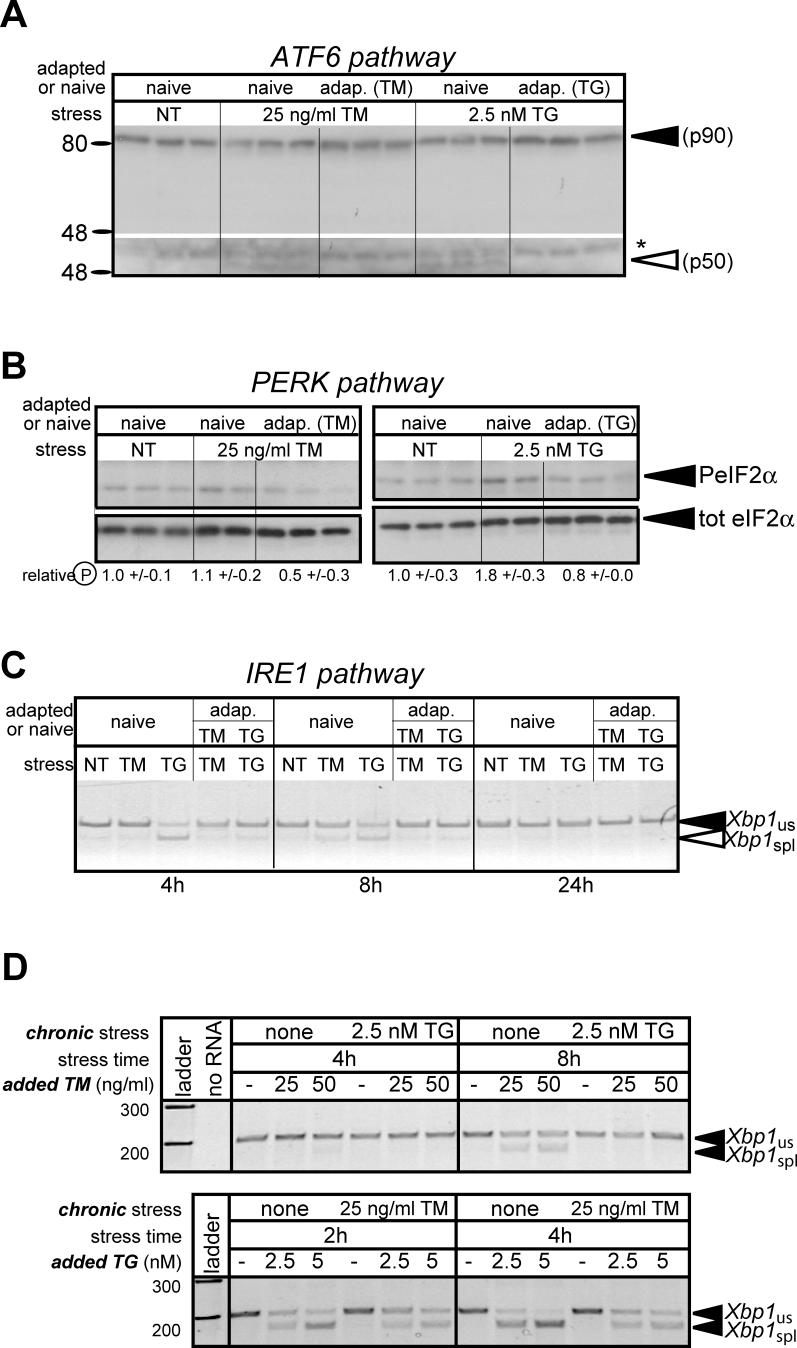
Adaptation Suppresses Further UPR Activation (A) Cells were cultured in triplicate for two passages in the continuous presence of 25-ng/ml TM or 2.5 nM TG (“adapted” cells), or vehicle (“naive” cells). TM or TG was then added to naive cells at the indicated concentrations, or re-added to adapted cells likewise. After 8 h (when ATF6α activation is most robust; see Figure 2A; also unpublished data), cellular lysates were collected and probed for expression of the active form of ATF6α by immunoblot. The top portion of the figure shows a lighter exposure (on this gel, the unglycosylated form of full-length ATF6α [p90] shifts only slightly relative to the glycosylated form), and the bottom portion shows a darker exposure of just the cleaved form, and a non-specific background band (marked with an asterisk [*]). Vertical hairlines are used for visual clarity (also in [B] and [C]). (B) Cells were treated as in (A), but lysates were taken at 8 h (for TM treatment) or 2 h (for TG treatment) after the addition of stressor, and probed by immunoblot for expression of either phosphorylated eIF2α (top portion) or total eIF2α (bottom portion). Average extent of eIF2α phosphorylation, normalized against naïve untreated cells and shown ± SDM, is given below each panel. (C) Cells were treated as in (A), and total RNA was harvested 4, 8, or 24 h after stressor addition or readdition, for RT-PCR amplification of *Xbp1* mRNA as in Figure 2B. (D) Cells adapted to chronic TG (top panel) or chronic TM (bottom panel), or cells passaged in parallel with vehicle alone (“none”), were treated with either TM (top panel) or TG (bottom panel) following adaptation in the opposite stressor. At the indicated intervals after exposure to the additional stressor, RNA was prepared and assessed for *Xbp1* splicing by RT-PCR as in Figure 2B.

We imagined three general possibilities for how BiP up-regulation, but not CHOP up-regulation, could be maintained as cells adapt to ER stress. In one scenario, cells become resistant to further UPR activation by virtue of improved protein folding and processing, and so activation of the proximal sensors of ER stress becomes suppressed; in this scenario, mechanisms downstream of proximal sensor activation are required for persistence of BiP up-regulation. An alternate possibility is that the UPR remains activated to a constant extent as long as cells are exposed to pharmacological inducers of stress, but downstream mechanisms suppress ongoing production of CHOP and GADD34. Finally, cells might selectively maintain activation of the proximal sensor (probably ATF6) upstream of BiP, but not of the sensor (probably PERK) upstream of CHOP. To distinguish among these possibilities, we compared UPR activation in cells that had been continuously exposed to TM or TG (“adapted cells”) and were challenged with fresh stressor, with UPR activation by TM or TG in passage- and confluence-matched vehicle-treated controls (“naive cells”). Although we could detect production of the active form of ATF6α, phosphorylation of eIF2α, and splicing of *Xbp1* mRNA in cells newly exposed to either TM or TG, these markers of UPR activation were not evident in adapted cells (Figure 4A–4C). Further, we found that adapted cells were not only resistant to continued exposure to the agent to which they had been adapted, but also displayed cross-tolerance against an independent inducer of ER stress, observed by diminished *Xbp1* mRNA splicing in TM-adapted cells in response to TG exposure, and vice versa (Figure 4D). In addition, cells adapted to ER stress induced by tetracycline-dependent overexpression of a secreted protein became resistant to a subsequent exposure to TG (Figure S7). Thus, activation of each of the three proximal sensors of ER stress is attenuated in adapted cells; and, because resistance to the pharmacological agent per se does not seem to occur (Figure S3), this attenuation is most likely a consequence of the improved protein folding and processing capacity that accompanies initial UPR activation. Although the UPR probably remains activated in adapted cells to an extent below the threshold of our ability to detect it, we conclude that improved protein folding capacity largely suppresses UPR activation and facilitates adaptation.

### Selective Instability of Pro-apoptotic mRNAs and Proteins

Adaptation of cells to pharmacologically-induced ER stress results in stable up-regulation of BiP (as well as other proteins that are involved in alleviating protein folding stress in the ER, including GRP94, calreticulin, and p58^IPK^—Figure S6 and unpublished data). However, production of CHOP protein fluctuates; it is induced most robustly upon initial exposure of cells to either TM or TG, and then its levels decline until re-addition of stressor (Figure S8). Over time, cells become less responsive to the stressor in terms of up-regulation of CHOP, to the point that, for either TM or TG, there are periods when adapted cells show no up-regulation of CHOP, but persistent up-regulation of BiP. The observation that activation of all three stress sensors declines despite persistent exposure to the pharmacological agent points to downstream mechanisms that cause CHOP production to fall commensurate with UPR activation, but allow BiP production to be maintained.

**Figure 5:**
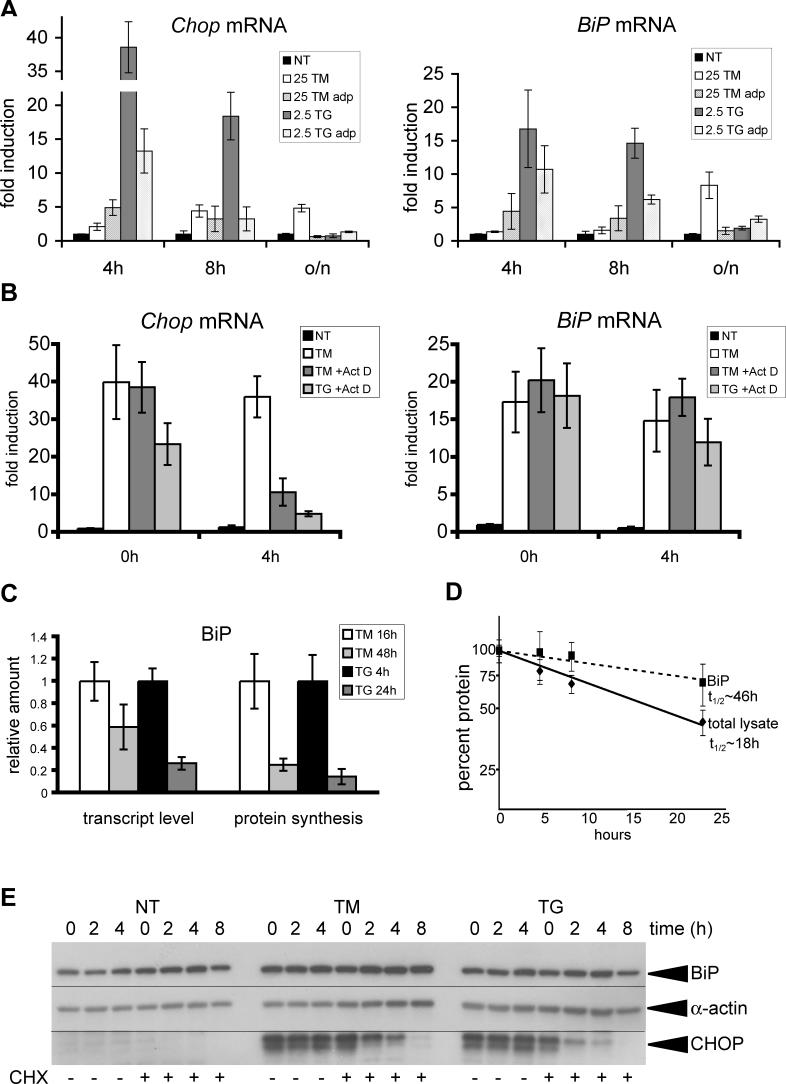
Pro-apoptotic mRNAs and Proteins Are Selectively Unstable (A) Naive or adapted (adp) cells were treated, or retreated, with TM or TG. After 4, 8, or 24 h of stress (for naive cells) or media re-addition (for adapted cells), total RNA was isolated and the expression of *BiP* and *Chop* was quantitated by real-time RT-PCR, normalizing against *18S* rRNA expression. Error bars represent means ± SDM from RNA isolated from three independent plates. (B) MEFs were treated with 25-ng/ml TM overnight or 2.5-nM TG for 4 h, and actinomycin D (Act D) was added as indicated to a final concentration of 5 μg/ml to prevent new mRNA synthesis. RNA was then harvested at 0 or 4-h time points (“time 0” followed 15 min of actinomycin treatment to allow for full effect), and expression of *Chop* and *BiP* mRNAs was measured by real-time RT-PCR as in (A). Error bars represent means ± SDM from replicate PCR reactions of a single experiment. (C) MEFs were grown for 48 h in media containing 10% of the normal amount of methionine and cysteine, and were treated either for the full 48 h or just the last 16 h with 25-ng/ml TG (or alternatively, cells were treated for 24 or 4 h with 2.5 nM TG). After treatment, three plates were immediately processed to harvest mRNA, and to the remaining three plates, ^35^S methionine/cysteine was added to a final concentration of 200 μCi/ml for 30 min, followed by cell lysis. Expression of *BiP* mRNA was assessed by real-time RT-PCR (“transcript level”), whereas the synthesis rate of BiP was measured by immunoprecipitation with an antibody recognizing the KDEL ER retention motif. SDS-PAGE and autoradiography revealed BiP expression, which was then normalized against TCA-precipitable counts from the immunoprecipitate supernatants, as a measure of total radioactivity input. Error bars are SDMs derived from independent readings of mRNA and protein synthesis from three plates each. (D) Untreated cells were subjected to steady-state labeling overnight with ^35^S Met/Cys. Cells were then chased in non-radioactive media containing excess methionine and cysteine, and either vehicle alone or 25-ng/ml TM for 0, 4, 8, or 24 h. The amount of radioactivity in the lysates was determined by TCA precipitation of aliquots, and was normalized against total protein concentration, whereas the amount of labeled BiP was assessed by immunoprecipitation. At each time point, three independent plates were harvested. The presence or absence of TM during the chase period did not significantly affect the measured half-life of either BiP or the lysate as a whole; therefore, each point represents input from replicate plates in both conditions. (E) MEFs were pretreated either for 4 h with 2.5 nM TG or overnight with 25 ng/ml TM to induce expression of CHOP. Cycloheximide (CHX) was then added as indicated at a final concentration of 50 μg/ml to block protein synthesis. Protein lysates were harvested 0, 2, 4, or 8 h after CHX addition and the expression of BiP, CHOP, and α-actin was probed by immunoblot.

One such mechanism to account for persistent BiP expression could be differential transcriptional activation of BiP versus CHOP. However, the inductions of both mRNAs shortly after exposure to TM or TG were quite comparable (Figure 5A, 4-h time point, compare *BiP* and *Chop* profiles). In contrast, after either naive or adapted cells had been exposed to stressor overnight, the steady-state level of *Chop* mRNA had in most cases fallen to levels comparable to untreated cells, whereas *BiP* mRNA remained elevated to a small but significant extent (Figure 5A, overnight time point). This observation raised the possibility that *BiP* mRNA might be substantially more stable than *Chop* mRNA, and so could contribute to the persistence of BiP expression even as UPR activation diminished. To test this hypothesis, we used actinomycin D (Act D) to inhibit new mRNA synthesis and assess *BiP* and *Chop* mRNA half-lives. Act D was added after pretreating cells with TM or TG to induce both *BiP* and *Chop* expression, and levels of *BiP* and *Chop* mRNAs were determined after four additional hours by quantitative real-time RT-PCR (Figure 5B). We found that *Chop* mRNA was degraded rapidly, with a half-life of approximately 2–3 h. In contrast, *BiP* mRNA showed little or no degradation over the same time period. In fact, mRNAs for *Atf4* and *Gadd34,* which are upstream and downstream, respectively, of *Chop* in a single gene expression axis, were also unstable, with similar degradation kinetics, whereas *Grp94* and *p58^IPK^* mRNAs were, like *BiP,* quite stable (Figure S9).

Preferential translation of *BiP* mRNA [60,61] could also contribute to the stability of BiP up-regulation. However, comparing BiP protein synthesis, measured by immunoprecipitation of ^35^S-labeled nascent chains, with mRNA levels, we found no evidence for preferential translation of BiP mRNA (Figure 5C). In contrast to translation, the stability of BiP protein compared to CHOP protein also appears to contribute to its persistent up-regulation; we found by pulse-chase analysis that BiP protein was, like its mRNA, quite stable. Although the half-life for cellular proteins as a whole was approximately 18 h, the half-life of BiP was approximately 46 h (Figure 5D). Lacking a suitable antibody for immunoprecipitation of CHOP, we measured CHOP protein stability instead by immunoblot after treatment of stressed cells with the protein synthesis inhibitor cycloheximide. This experiment confirmed the stability of BiP protein, and also demonstrated that CHOP was degraded with a half-life of 4 h or less (Figure 5E). ATF4 and GADD34 proteins were similarly unstable, whereas GRP94 and p58^IPK^ proteins were, like BiP, quite stable (unpublished data). Thus, in attempting to determine how the expression of BiP and other proteins that facilitate protein folding and processing could be persistently up-regulated even as the expression of pro-apoptotic proteins like CHOP diminished, we observed marked differences in mRNA and protein stability, and not in upstream sensor activation, nor transcriptional activation, nor translation. Therefore, we conclude that the degradation rates of these mRNAs and proteins are important in generating the UPR that accompanies adaptation, wherein CHOP expression is modest and transient.

### An Adapted Phenotype Is Recapitulated in Cells with Compromised ER Quality Control

Pharmacological induction of mild ER stress has revealed several salient features of the pro-adaptive UPR that arise as a consequence. Most importantly, these experiments have illustrated that a cell population that has adapted to ER stress can maintain an activated UPR without displaying some of the classical hallmarks of UPR activation such as up-regulation of downstream genes like *Chop*.

To determine whether these insights apply to adaptation to non-pharmacological ER stress, we turned to MEFs derived from mice homozygous for genetic ablation of the UDP-glucose glycoprotein glucosyltransferase *(Uggt1)* gene. UGGT1 catalyzes glucose re-addition to misfolded proteins in the calnexin/calreticulin cycle, and so is thought to be an important component of misfolded protein recognition and the ER quality control machinery [62]. UGGT1 deficiency leads to embryonic lethality approximately 12.5 dpc (D. T. Rutkowski, S. M. Arnold, R. J. Kaufman, unpublished data); however, MEFs were isolated that showed no apparent gross defects in protein biogenesis nor a substantial growth defect [63]. *Uggt1^−/−^* cells have been shown to be defective in the processing of certain glycoproteins through the calnexin/calreticulin cycle [63], leading to the conclusion that, while *Uggt1* is clearly essential for development, its presence is not strictly required for cell viability and proliferation despite the fact that its deletion results in the production of misfolded proteins in the ER. We therefore hypothesized that *Uggt1* deficiency represents a genetic form of chronic stress, and that *Uggt1*-deficient MEFs have been able to adapt to the ER stress burden. If this hypothesis is true, then *Uggt1*-deficient cells should display at least in part the hallmarks of an adapted phenotype.

**Figure 6:**
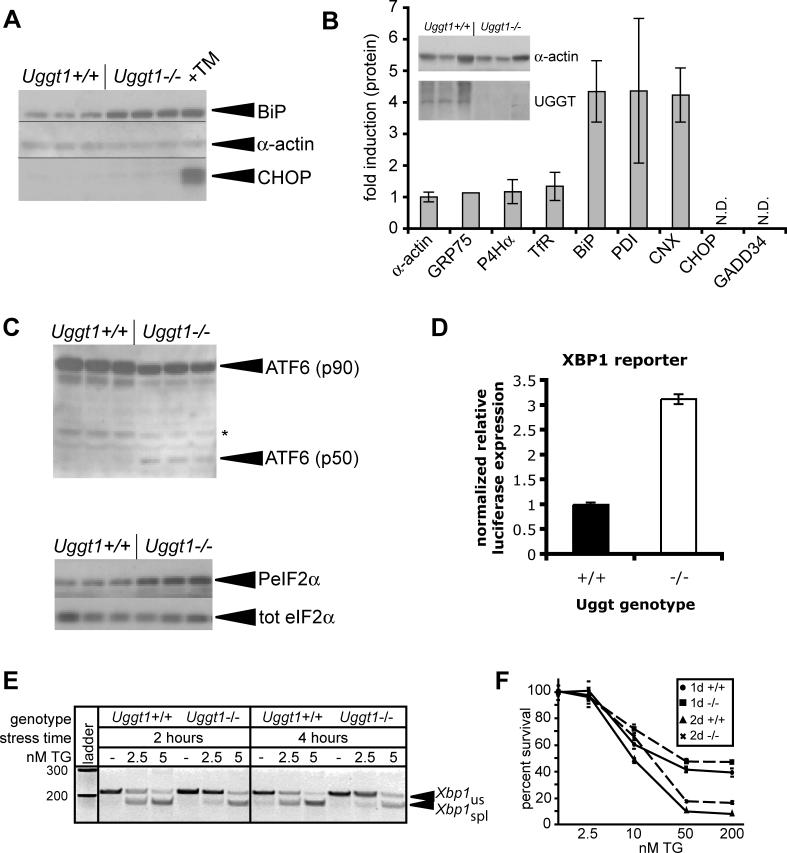
*Uggt1* Deficiency Recapitulates an Adapted Phenotype (A) Lysates were prepared from three independent plates of either *Uggt1^−/−^* MEFs or wild-type counterparts, or from one plate of wild-type MEFs treated with 1-μg/ml TM overnight. BiP, α-actin, and CHOP expression were probed by immunoblot as in Figure 3B. (B) Lysates were prepared from either *Uggt1^−/−^* MEFs or wild-type counterparts. Immunoblot for α-actin and UGGT1 (inset) from three separate plates confirmed their identities. The lysates were then probed by immunoblot for expression of α-actin, the mitochondrial chaperone GRP75, the collagen-specific ER chaperone prolyl-4-hydroxylase-α (P4Hα), the membrane-localized transferrin receptor (TfR), BiP, PDI, calnexin (CNX), CHOP, or GADD34. Expression was normalized against α-actin. Error bars represent means ± SDM from at least three measurements, except for Grp75 for which data could only be reliably quantitated from one measurement because of high background. CHOP and GADD34 were not quantitatively detected in either lysate. (C) Lysates prepared as in (A) were analyzed for production of the cleaved form of ATF6α (top panel) or the phosphorylated form of eIF2α (bottom panel) by immunoblot. The band marked as p50 comigrates with a band induced by high TM treatment in wild-type cells (unpublished data). (D) Wild-type and *Uggt1^−/−^* MEFs were cotransfected with an XBP1-dependent luciferase reporter and a constitutive β-galactosidase reporter. Lysates were analyzed for luciferase expression 24 h after transfection, normalized against β-galactosidase expression. Error bars represent means ± SDM from three independent plates. (E) Wild-type and *Uggt1^−/−^* MEFs were treated with 2.5 or 5 nM TG for 2 or 4 h, and *Xbp1* splicing was assessed as in Figure 2B. (F) Wild-type (solid lines) and *Uggt1^−/−^* (dashed lines) MEFs were plated on 96-well plates and treated with increasing concentrations of TG, for either 1 or 2 d. Cell proliferation was estimated by an MTT assay, with each genotype normalized against untreated cells of that genotype. The MTT assay measures mitochondrial reduction of a tetrazolium dye, and is a measure of cell viability and proliferation.

*Uggt1^−/−^* MEFs showed a specific up-regulation of ER chaperones. Expression of BiP, PDI, and calnexin was elevated in *Uggt1^−/−^* cells relative to wild-type counterparts, whereas the expression of a sampling of non-ER proteins and non-chaperone proteins of the endomembrane system was comparable in both genotypes (Figure 6A and 6B). Notably, we did not detect quantitative expression of CHOP or GADD34 proteins in either cell type (Figure 6A and 6B). Electron microscopic analysis of wild-type and *Uggt1^−/−^* cells did not reveal any obvious abnormalities in ER morphology in the knockout cells (unpublished data).

To determine whether elevated chaperone expression was a consequence of UPR activation in *Uggt1* ^−/−^ cells, we monitored the activation status of the ER stress sensors ATF6α, PERK, and IRE1. We detected by immunoblot both the cleaved form of ATF6α and the phosphorylated form of eIF2α in *Uggt1^−/−^* cells even in the absence of an exogenous ER stress-inducing agent (Figure 6C). Although *Xbp1* mRNA splicing was not detected by RT-PCR in the absence of exogenous stress (see Figure 6E), activation of an XBP1-dependent luciferase reporter [27] suggested that IRE1 was also activated in *Uggt1* ^−/−^ cells (Figure 6D). Consistent with these results, we also found mRNAs of UPR target genes to be elevated both in *Uggt1* ^−/−^ MEFs, and in total RNA preparations from whole day 10.5 embryos (Figure S10). Thus, *Uggt1* deletion appears to lead to modest UPR activation in these MEFs. As in pharmacologically adapted cells, *Uggt1^−/−^* cells show apparent selective up-regulation of pro-survival proteins including ER chaperones, but not pro-apoptotic proteins such as CHOP and GADD34.

Because cells adapted to pharmacological stress show cross-tolerance (Figure 4D), we reasoned that *Uggt1^−/−^* cells, if they are indeed adapted to stress, might display resistance to chemical stress. To test this hypothesis, we treated cells of both genotypes with low concentrations of TG and monitored UPR activation by *Xbp1* splicing. By this measure, *Uggt1^−/−^* cells were relatively resistant to ER stress (Figure 6E). This parallel supports the notion that *Uggt1^−/−^* cells have adapted to chronic stress. The resistance to stress as seen at the molecular level was reflected in a slight but significant enhancement of proliferative capacity in *Uggt1^−/−^* cells in the presence of an exogenous ER stressor (Figure 6F). Thus, while it might have been expected a priori that *Uggt1^−/−^* cells would be more sensitive to ER stress because of a compromised quality control system, the resistance of these cells is consistent with their having achieved an adapted state that parallels the state achieved in cells adapted to pharmacological stress. These results also support the notion that up-regulation of ER chaperones that occurs as a consequence of chronic stress exposure is protective.

### A mathematical model for adaptation

The goal of the experiments presented in this work was to understand how the UPR could selectively become a program for allowing cells to survive and adapt to ER stress. Toward that end, we have established a cell culture system that has demonstrated that cell death is not an obligate endpoint of persistent ER stress. By exposing cells to low concentrations of pharmacological inducers of ER stress, we have shown that mild ER stress is accompanied by full UPR activation, but the response is qualitatively distinct in its effects on gene expression from a terminally activated UPR. We have also found that the most salient features of this adaptive response are important components of the survival response to another persistent ER stress, induced by genetic disruption of ER quality control.

**Figure 7:**
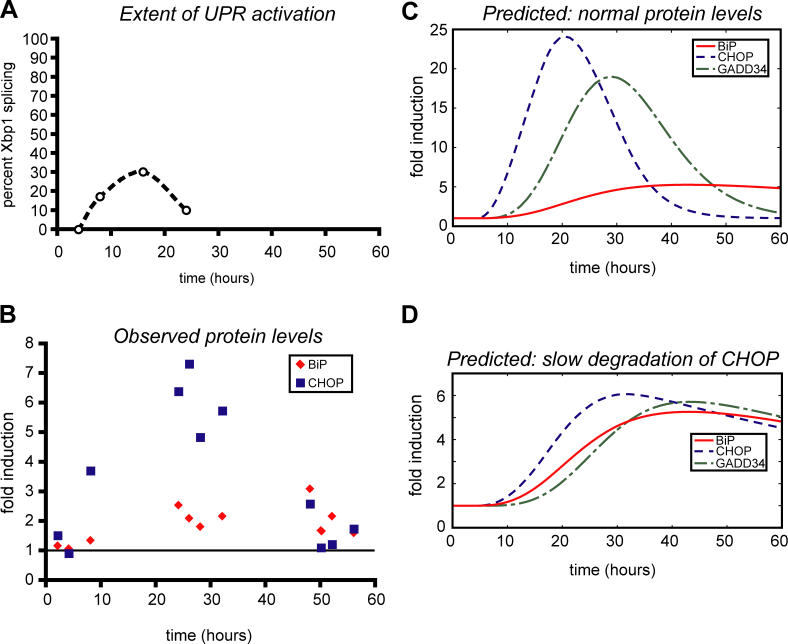
Rapid Degradation of CHOP and GADD34 mRNAs and Proteins Allows Their Expression to Be Down-Regulated as Cells Adapt (A) *Xbp1* mRNA splicing during a 24-h time course of TM treatment (25 ng/ml) was quantitated and used to approximate the stress level in cells at given times (taken from Figure 2B and unpublished data). These data were used as the basis for the stress input in a mathematical model describing the production of BiP, CHOP, and GADD34 mRNAs and proteins. The dashed line is used only to provide a visual aid for the trend of splicing over the time course. (B) The relative expression of CHOP and BiP protein was quantitated by immunoblot from cells exposed to 25-ng/ml TM over a 48-h time course (the media and stressor were refreshed at 24 h), normalized against untreated cells (data taken from an experiment, not shown, similar to Figure 3A and 3C). (C) and (D) A mathematical model used experimental data (such as that obtained from Figures 3 and 5) to derive production and degradation rates for ATF4, CHOP, and GADD34, and ATF6 and BiP, and was used to test the effect of altering the stabilities of components within the ATF4-CHOP-GADD34 axis on the responsiveness of these components to the stress level. “C” shows the model-predicted expression of these proteins using their experimentally derived degradation rates, while “D” illustrates the changes in CHOP and GADD34 protein levels brought about by making the degradation rate of CHOP protein comparable to that of BiP protein. Note the change in y-axis scale between panels “C” and “D”; expression of BiP does not change between these panels. See text and Protocol S1 for further details.

Our data suggest that the key distinction between expression of ER chaperones such as BiP and GRP94, and expression of ATF4, CHOP, and GADD34, is their dramatically different mRNA and protein stabilities, which leads to differential expression of the former but not the latter as cells adapt to stress. However, it is not currently possible to *selectively* alter the stabilities of individual *endogenously expressed* mRNAs or proteins for which no specific degradation machinery has yet been identified. Therefore, we created a simple mathematical model to describe the expression patterns of BiP, and of CHOP and GADD34, derived from our experimental data and based on the following assumptions: (1) that *Xbp1* mRNA splicing serves as a reporter for the level of stress in the ER, as it has been in previously published literature [44] (Figure 7A); (2) that both ATF4 and the active form of ATF6 would be produced to a level commensurate with the stress burden and both proteins would have similar degradation rates (a simplifying assumption); and (3) that the expression of all proteins in the model would depend solely upon uniform rates of production and degradation (another simplifying assumption). Thus we produced a model in which the degradation rates of proteins and mRNAs in the ATF6-BiP axis and the ATF4-CHOP-GADD34 axis could be manipulated and the effects on the expression of these proteins determined (Figure 7; see also Protocol S1 for a more thorough discussion of the generation of the model and its manipulation). The resulting model predicts that the relative instability of ATF4, CHOP, and at least one target of CHOP (GADD34) ensures that the expression of proteins in this gene expression axis will be fairly responsive to the actual stress level experienced by a cell at a given time. In contrast, the elevated expression level of BiP would extend significantly beyond the period of stress, resulting in a scenario wherein BiP remains up-regulated, but CHOP and GADD34 do not. This modeling fairly well predicts the measured outcome derived from analysis of the expression levels of BiP and CHOP, and suggests that the rapid degradation of ATF4, CHOP, and GADD34 is alone sufficient to mirror the experimental outcome (Figure 7B and 7C). If one or more of the components of this ATF4-CHOP-GADD34 axis were stable to the same extent as BiP mRNA or protein, production of GADD34 and other targets of CHOP would persist long after the stress burden was alleviated (Figures 7D, and S11–S14), and so the rapid responsiveness of this pathway demands rapid degradation of all of its components. Assuming that one or more of the targets of CHOP is pro-apoptotic, cell death would be more strongly favored even by mild UPR activation. This finding raises the possibility that the degradation rates of these components might vary from cell-type to cell-type, and so contribute to differing sensitivities to ER stress and to the threshold at which adaptation can be chosen over death.

### A Possible Mechanism of Adaptation

Perhaps the most illuminating result of this work is that it suggests a rationale to explain how the UPR can be a predominantly adaptive pathway under certain circumstances. The central feature of an adaptive response to ER stress appears to be maintenance of expression of proteins that facilitate survival, in particular ER chaperones, without persistence of pro-apoptotic proteins such as CHOP and GADD34. In principle, this result might have been most easily rationalized by invoking selective activation of ATF6α, which is thought to control the transcriptional regulation of BiP, GRP94, calreticulin, and other ER chaperones [21], with deactivation of PERK signaling, which is thought to be the principle driver of CHOP expression [21,22]. However, selective activation of one or more proximal sensors of ER stress has not been convincingly demonstrated experimentally here or elsewhere (the presence of selective activation has typically been inferred by an apparent failure to up-regulate specific UPR target genes), and indeed, we have been unable to produce selective activation in response to any of a number of different stresses (this work and unpublished data). More likely, the pathways of the UPR are structured in such a way that survival is favored as an intrinsic outcome even when all pathways are activated, and that the selectivity of expression achieved at the protein level is a consequence of the UPR hierarchy and not of selective activation.

**Figure 8:**
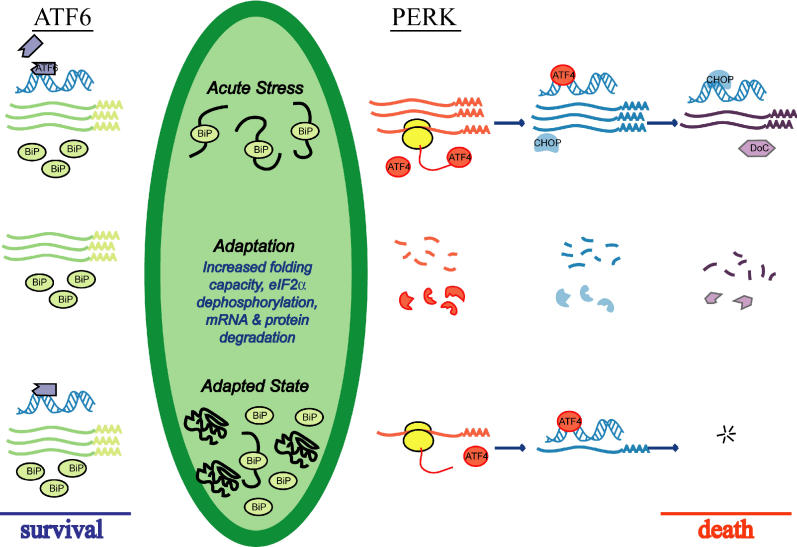
Model for Generation of an Adaptive Response to Chronic Stress The initial exposure of cells to ER stress leads to activation of all three proximal sensors of stress (only the ATF6 and PERK pathways are shown here) and up-regulation of UPR target genes. However, prolonged exposure to mild stress allows for adaptation, when selective degradation of mRNA and protein attenuates expression of CHOP and its downstream targets (DoCs), but not of ER chaperones like BiP. Increased BiP levels in the ER facilitate adaptation, both by assisting protein folding, and probably by binding to, and thereby inhibiting activation of, the proximal sensors. An adapted state might be characterized by low-level activation of the sensors, that is sufficient to maintain elevated BiP protein levels, but not of CHOP or GADD34. See text for further details.

Our results allow us to propose a model for adaptation to ER stress (Figure 8). As the cell first experiences ER stress, BiP dissociation activates IRE1, PERK, and ATF6, initiating the signaling cascades that comprise the UPR [4,64]. When the stress is robust and persistent, the layers of negative regulation acting on the response are insufficient to thwart the pro-apoptotic program. CHOP, an important pro-apoptotic protein in its own right, and also presumably a sentinel for the activation of pro-apoptotic cascades in general, is expressed at levels too high to be down-regulated, and apoptosis occurs as a consequence. However, when the stress sensed by the cell is milder, the overall level of activation of PERK, IRE1, and ATF6 is considerably less. Because ATF4, CHOP, and GADD34 are quite unstable at both the mRNA and protein levels, changes in the expression of these proteins are necessarily short-lived in the absence of a positively perpetuating stress signal. Any improvement in the protein folding capacity of the cell, by either increased production of chaperones such as BiP or by loss of the potency of the stressor, will have the effect of suppressing activation of the proximal stress sensors [65] and causing the levels of CHOP protein and its targets to decay along with the attenuation of UPR activation. Further, the PERK pathway is subject to negative regulation at multiple levels: GADD34 and CreP promote eIF2α dephosphorylation [41,43,53], whereas p58^IPK^ is thought to inhibit PERK [66,67]. Therefore, although the stress on the organelle might persist and PERK may continue to catalyze a small amount of eIF2α phosphorylation, the amount of ATF4 produced, and further downstream, of CHOP and GADD34, is destined to be small. Conversely, although ATF6 is also activated only to a minor extent by mild ER stress, its effect on the expression of downstream proteins should be more long lived. Even as the stress is partially resolved and ATF6 activation is diminished (both by an improvement in protein folding and a rapid turnover of ATF6α [32,68,69]), a minor and even transient elevation in the levels of activated ATF6 over baseline is bound to have a lasting effect on expression of ER chaperones such as BiP because of the stability of these genes at both the mRNA and protein levels. Because of these properties in the adaptive response, changes in the expression of UPR target proteins do not correlate in a predictable way with the activation of PERK, IRE1, or ATF6 [70]. It seems likely that this characteristic of the UPR might also explain the long-observed preconditioning phenomenon, whereby transient exposure to one form of stress protects cells against subsequent stresses (e.g., [71,72]). The consequence of transient exposure should be the persistent up-regulation of proteins that facilitate survival without the induction of apoptosis, so that the cell is better able to withstand the subsequent stress.

An additional influence that might act to facilitate adaptation is the placement of ATF4, CHOP, and GADD34 in a multistep regulatory cascade. At both the mRNA and protein levels, GADD34 is not as readily up-regulated as CHOP by low doses of stress (Figures 3 and S15). A characteristic of genetic and signaling networks is that successive steps in the network require a higher stimulus for activation, and their responsiveness becomes increasingly binary [73–75]. ATF4, CHOP, and GADD34 likely form a network of this sort, and it is conceivable that the placement of these proteins into such a network allows a linear gradient of stress to be converted into a binary life-or-death signal. Stress that falls below the necessary threshold should fail to elicit production of CHOP-dependent death effectors. In support of this idea, we found that under conditions of mild stress, *Gadd34* mRNA was, in most cases, not up-regulated to a significant extent even when *Chop* mRNA up-regulation was observed (Figure S15). In contrast, the expression of BiP and other ER chaperones is thought to lie directly downstream of ATF6, leaving less of an opportunity for the stress signal to dissipate en route to chaperone production. Although it is difficult to test this hypothesis directly in a metazoan cell or, at this point, mathematically, it is noteworthy that GADD34 protein production seems to have a very sharp threshold for induction, and to correlate very closely with the survival or death decision experienced by the cell population. It is also noteworthy that most of the pro-survival genes up-regulated as a consequence of PERK activation are probably regulated directly by ATF4, whereas the pro-apoptotic activity of ATF4 depends on the CHOP-induced transcriptional effects on downstream genes. Thus, the ATF4-dependent pro-survival genes lie one step more proximal to the activation status of PERK, and so, if PERK remains activated to a very small extent, are more likely to remain elevated as a consequence of their placement in the genetic network—particularly if they, like BiP and GRP94, are stable at the protein and mRNA levels.

### Conclusions and Perspectives

An important conclusion of this work is that the adapted state is best characterized by changes in the protein composition of adapted cells, rather than by changes in the activation status of UPR transducers or in changes in genomic expression. Proteomic characterization, although technically challenging, will likely help define how a cell responds to chronic stresses of different types. Temporal regulation of an adaptive response can potentially confound attempts to define whether or not the UPR is activated by simply monitoring activation of one component, such as *Xbp1* splicing or production of CHOP, since the adaptive response is, in its outcome, qualitatively distinct from the simple experimentally induced robust UPR.

This work was necessarily limited to exploring a very small subset of the most proximal activation events in governing the choice between cell life and death, and is meant only to provide a general framework for how these decisions are made. Evolution would seem to favor a stress-sensing pathway that was designed to commit to death only when all protective mechanisms had been overwhelmed. In this view, there is no single apoptotic pathway that represents a commitment to death; rather, the cell would depend on input from all of them. Stronger stresses would prevent the attenuation of apoptotic signals, with each pathway amplifying the commitment to death.

## Materials and Methods

### Cell culture.

MEFs were maintained in DMEM containing 4.5-g/l glucose (Invitrogen catalog 11995–065; Carlsbad, California, United States) supplemented with 10% FCS, L-glutamine, penn/strep, amino acids, and non-essential amino acids (Invitrogen), at 37 °C in a 5% CO_2_ incubator. For *Perk^−/−^* and matched wild-type cells, βME was added to a final concentration of 50 μM. *Uggt1^−/−^* and *Uggt1^+/+^* cells were generated as described [63].

For all TM and TG treatments, 1,000× stocks of stressor were made in DMSO. For nontreated cells, DMSO alone was added to a final concentration of 0.1%. Cells were plated at a concentration of 1.5–2 × 10^5^ cells/well in six-well dishes, 4–5 × 10^5^ cells/60-mm dish, or 1 × 10^6^ cells/10-cm dish, and allowed to rest overnight before application of stress. Stressor stocks were dispensed into single-use aliquots and frozen, to maintain consistency. For long-term stress experiments (>24 h), the medium was removed and replaced with fresh media containing the diluted stressor or DMSO alone every 24 h, including immediately after trypsinization. For adaptation, cells were typically cultured for at least two passages in the presence of stressor, refreshed every 24 h. TM, TG, and cycloheximide were from EMD Biosciences (San Diego, California, United States). Actinomycin D was from Sigma (St. Louis, Missouri, United States).

Cell proliferative rate was measured by trypsinizing cells, resuspending in an equal volume of complete media, and diluting into IsoFlow Sheath Fluid (Beckman Coulter, Fullerton, California, United States), followed by counting on a Coulter Z1 particle counter. Each sample was counted twice. MTT reduction was measured using the CellTiter 96 Aqueous One kit (Promega, Madison, Wisconsin, United States) according to the manufacturer's instructions.

### Protein analysis.

MEF protein lysates were prepared in either 1% SDS, 100 mM Tris (pH 8.9) followed by vigorous boiling, or in 1% Triton X-100, 100 mM Hepes (pH 7.4), 100 mM NaCl. The lysis method had no bearing on the results obtained. For immunoblots, lysates were separated on Criterion Tris-HCl gels (Bio-Rad, Hercules, California, United States), typically about 5 μg of protein per lane, and transferred to PVDF membrane (GE Healthcare, Little Chalfont, United Kingdom) overnight in a transfer tank. Blots were processed and developed using ECL Plus, following the manufacturer's protocol (GE Healthcare). Blots were exposed to Hyperfilm ECL (GE Healthcare). Blotting steps were carried out at room temperature. Preflashing film had no effect on results obtained. Antibody specificity was verified by overexpression controls or use of knockout cell lines. Antibodies were supplied by BD Biosciences (BiP, used at 1/5,000; San Diego, California, United States), Cell Signaling Technology (Phospho-PERK 1/1,000; Beverly, Massachusetts, United States), MP Biochemicals (α-actin, 1/500,000; Solon, Ohio, United States), Santa Cruz Biotechnology (CHOP, GADD34, and ATF4, all 1/200; Santa Cruz, California, United States), Nventa Biopharmaceuticals (KDEL and UGGT1, 1/500; and PDI 1/20,000; San Diego, California, United States), Invitrogen (phosphorylated elF2α, 1/1000), and Zymed (transferrin receptor, 1/500; Invitrogen). For ATF6α immunoblot, lysates were prepared as described [21], and 100 μg of protein per lane was run on a 7.5% Tris-HCl gel (Figure 2A) or a 10.5%–14% Tris-HCl gel (Figure 4A) and probed with anti-ATF6α [11]. In all cases, blots shown together were taken from single membranes by slicing the membrane and probing each slice individually. Hairlines on images indicate where two images were electronically spliced together.

For analysis of protein degradation by ^35^S incorporation, cells were incubated overnight in an 80/20 mixture of Met/Cys-free media/complete media, along with 100-μCi/ml ^35^S-Met/Cys labeling mix (ProMix; GE Healthcare). Cells were then washed twice in complete media and chased in complete media supplemented with 5 mM cold Met/Cys in the presence or absence of 25-ng/ml TM. Cells were washed on ice twice in cold PBS, then lysed in 100 mM Tris (pH 8.9), 1% SDS, followed by boiling. Total protein concentration of the lysate was measured using the Bio-Rad DC protein assay kit. Total radioactivity was measured by TCA (trichloroacetic acid) precipitation on a filter [20]. Equal volumes of lysate were diluted into ten volumes of IP buffer (1% Triton X-100, 100 mM Hepes [pH 7.4], 100 mM NaCl), precleared for 30 min with Protein A-agarose (Pierce Biotechnology, Rockford, Illinois, United States), and the supernatants incubated overnight with anti-KDEL (5 μg/ml final; Nventa Biopharmaceuticals) and Protein A-agarose. For determination of the rate of protein synthesis (Figure 5C), cells were incubated for 48 h in 10/90 complete media/Met-Cys- media, with 25-ng/ml TM added either at the beginning of the time period, or after 32 h. ProMix was then added directly to the media to a final concentration of 200 μCi/ml for 30 min, followed by lysis and immunoprecipitation as described above. Alternatively, cells were treated with 2.5 nM TG for 4 or 24 h in the same general way. Aliquots of immunoprecipitation supernatants were subjected to TCA precipitation [20].

For luciferase assays, cells were transfected using FuGene6 (Roche, Indianapolis, Indiana, United States), with plasmid pcDNA CMV-lacZ and 5× UPRE-luciferase [27]. Luciferase was measured using the Dual Light assay kit (Tropix) according to the manufacturer's instructions.

Two-dimensional SDS-PAGE was performed by lysing cells in hypotonic buffer (10 mM Tris pH 7.5) and sonicating, and isolating the 100,000×*g* membrane pellet by differential centrifugation. The pellet was solubilized in 7 M urea, 2 M thiourea, 2% CHAPS, 2% amidosulfobetaine (ASB-14), 2 mM tributylphosphine (TBP), 0.2% pH 3–10 ampholytes (Bio-Rad), and 0.1% bromophenol blue. Lysates were separated in the first dimension on pH 3–10NL Ready-Strips (Bio-Rad) and in the second dimension on 10.5–14% Tris-HCl Criterion gels (Bio-Rad). Gels were stained using SyPro Ruby (Bio-Rad) and documented using a Typhoon 9400 Phosphorimager (GE Healthcare).

### RNA analysis.

Total cellular RNA was isolated either by RNeasy (Qiagen, Valencia, California, United States) or Trizol reagent (Invitrogen). For *Xbp1* RT-PCR, the Titan One-Tube RT-PCR kit (Roche) was used along with primers flanking the *Xbp1* intron to amplify both spliced and unspliced *Xbp1*. Real-time RT-PCR was performed by first generating cDNAs using the iScript kit (Bio-Rad), which uses random primers. cDNA was then diluted (the extent varied based on the amount of starting RNA) and amplified by PCR in an iCycler (Bio-Rad) using iQ SYBR Green Supermix (Bio-Rad). For each primer set used, a dilution series of cDNA was first established to verify that amplification efficiency was near 100%, and to establish the linear range for the primer pair. Reaction products were separated by DNA electrophoresis to confirm that the amplified product was the correct size. For real-time reactions, a melt-curve analysis was performed at the end of the reaction to confirm the amplification of a single product with no primer dimers. Real-time reactions also included a control where no reverse transcriptase was included in the cDNA synthesis reaction, to exclude amplification of genomic DNA. Each primer pair was designed to span an intron. Primer pairs used were: *18S* rRNA: cgcttccttacctggttgat and gagcgaccaaaggaaccata; *Chop:* ctgcctttcaccttggagac and cgtttcctggggatgagata; *Gadd34:* gagattcctctaaaagctcgg and cagggacctcgacggcagc; *β-actin:* gatctggcaccacaccttct and ggggtgttgaaggtctcaaa; *BiP:* catggttctcactaaaatgaaagg and gctggtacagtaacaactg; *Grp94:* aatagaaagaatgcttcgcc and tcttcaggctcttcttctgg; *Atf4:* atggccggctatggatgat and cgaagtcaaactctttcagatccatt; *p58^IPK^:* tcctggtggacctgcagtacg and ctgcgagtaatttcttcccc; and *Uggt1:* gctttggtgtgaaacgtg and cagtttgggctccttagtc. Although the absolute extent of up-regulation for any gene varied somewhat from experiment to experiment, the trends of their expression changes were consistent.

### Electron microscopy.

MEFs were prepared for electron microscopy (EM) by fixing adherent cells on the plate using 2.5% glutaraldehyde in Sorensen's buffer. Samples were post-fixed in 1% osmium tetroxide for 1 h, rinsed, and the cells scraped, pelleted, and stained in saturated uranyl acetate. Samples were rinsed again, dehydrated in an EtOH series, and infiltrated with Spurrs resin. Samples were examined using a Philips CM100 electron microscope at 60 kV (Philips Medical Systems, Andover, Massachusetts, United States). Images were recorded digitally using a Kodak 1.6 Megaplus camera system operated using AMT software (Advanced Microscopy Techniques Corp., Danvers, Massachusetts, United States). During microscopic examination, nucleated cells were randomly chosen for documentation, with the treatment, if any, of the cells, not known at the time of observation; and regions of ER within each cell were randomly chosen for more detailed imaging. For scoring, printed images were randomized with no visible identifying information and binned into groups based on ER morphology. Pooled images from two separate experiments were analyzed.

## Supporting Information

Figure S1 Mild TG Treatment Has Subtle Effects on ER Morphology MEFs treated with 100 nM TG overnight, or 2.5 nM TG overnight or chronically, were prepared for transmission electron microscopy as in Figure 1C. All images are 25,000×. Bar represents 500 nm.
(1.6 MB TIF)

Figure S2 Recovery of Proliferative Capacity Accompanies Adaptation Cells that had been chronically (~1 mo) exposed to 2.5 nM TG or 25-ng/ml TM were plated in replicate, as were cells passaged for the same interval in vehicle alone. To the previously untreated (“naive”) cells, 2.5 nM TG or 25-ng/ml TM was added for 2 d, whereas adapted cells (“adap”) were continued under the same stress conditions. Cell number was determined and quantitated as for Figure 1D, and is expressed as a percentage relative to the starting cell number, which was comparable for all five conditions.
(149 KB TIF)

Figure S3 Adapted Cells Remain Pharmacologically Sensitive to TM and TG (A) Naive or TM-adapted cells were challenged with 25-ng/ml TM, or 1-μg/ml TM as a positive control, and were then metabolically labeled in parallel for 4 h with ^3^H mannose or ^35^S methionine, after either 1 h or 20 h of TM treatment. Protein lysates were collected and an aliquot saved for determination of total protein concentration, whereas the remainder was subject to TCA precipitation. After normalization of incorporated ^35^S or ^3^H against total protein concentration, the relative amount of ^3^H incorporation relative to average ^35^S incorporation was determined. The extent of ^3^H incorporation for untreated cells was set to 100%. The initial effect of TM is greater in adapted cells, likely as a consequence of additive effects of the drug upon re-addition. Error bars represent means ± the standard deviation of the mean (SDM) from three independent plates. Only one plate was used for the high-TM positive control.
(B) Naive or TM-adapted MEFs were continued in either vehicle alone (→NT) or 25-ng/ml TM-containing media (→TM) and  incorporation of ^3^H mannose 24 h after stressor addition was measured as in (A). Thus the inhibition of glycosylation in adapted cells compared to adapted cells from which the stress was withdrawn is similar to that seen in nave cells newly exposed to TM, compared to untreated cells.
(C) Ca^2+^ responses were induced in untreated mouse embryonic fibroblasts (“NT”), as well as those exposed to 2.5 nM TG overnight (“TG”), using 1 μM TG. The break in each trace shows where TG was added after baseline data collection. The panel illustrates one experiment of the three used in the statistical analysis. The two groups, i.e., NT and TG, showed no significant difference in their Ca^2+^ response (*p* = 0.5).
(450 KB TIF)

Figure S4 Mild ER Stress Does Not Produce Extensive Apoptosis MEFs were treated for 72 h in the indicated concentrations of TM. Both floating and adherent cells were pooled and stained with Annexin V conjugated to EGFP, followed by FACS analysis. “C” and “D” represent arbitrary thresholds for annexin V positivity.
(820 KB TIF)

Figure S5 Low Concentrations of TM Have Only a Slight Effect on Translation Inhibition MEFs were treated for the indicated times with 25- or 1,000-ng/ml TM, or 2.5 or 100 nM TG. Cells were then briefly rinsed in Cys/Met-free media, and then incubated for 10 min in Cys/Met-free media containing the appropriate stressor plus ^35^S Cys/Met. Cells were then immediately rinsed and lysed, and the cell lysates were subjected to TCA precipitation on a filter followed by scintillation counting. The radioactivity precipitated was normalized against the measured protein concentration of the lysate. Error bars represent means ± SDM from three precipitations. The recovery of protein synthesis likely occurs as a consequence of GADD34-mediated eIF2α dephosphorylation.
(531 KB TIF)

Figure S6 BiP And GRP94 Are Up-Regulated in Wild-Type and *Ire1* ^−/−^ MEFs during Chronic Stress Wild-type or *Ire1* ^−/−^ MEFs were treated with TM as indicated, and lysates prepared for immunoblot 1, 3, or 7 d after the beginning of treatment. BiP and GRP94 were probed by an antibody recognizing the KDEL ER retention motif, with α-actin shown as a loading control.
(507 KB TIF)

Figure S7 Adaptation to Stress Induced by Overexpression of a Secreted Protein Produces Cross-Tolerance (A) CHO cells stably transfected with a tetracycline-responsive plasmid for production of blood coagulation Factor VII were treated with increasing concentrations of tetracycline as indicated. Cellular lysates were probed for expression of BiP, α-actin, or Factor VII. Control cells stably transfected with empty vector showed no changes in BiP expression, nor production of Factor VII, upon treatment with any dose of tetracycline.
(B) Splicing of *Xbp1* mRNA was determined in Factor VII–expressing cells after treatment with 10- or 25-ng/ml tetracycline as indicated. Note that 10 ng/ml is insufficient to elicit UPR activation, whereas 25 ng/ml results in *Xbp1* splicing.
(C) Factor VII-expressing cells were continuously cultured in 10- or 25-ng/ml tetracycline. Cells were then changed into media without tetracycline and allowed to rest, and 10 nM TG or vehicle was added as indicated. *Xbp1* mRNA splicing was determined by RT-PCR. Note that resistance to TG is seen only for adaptation in 25-ng/ml tetracycline, which activates the UPR, and not for 10-ng/ml tetracycline, which does not.
(1.0 MB TIF)

Figure S8 Up-Regulation of CHOP Protein Is Less Robust and Less Persistent in Adapted Cells Naive or adapted cells were challenged with addition of TM or TG as in Figure 4, and protein lysates taken 8 or 24 h after stress addition were probed for expression of CHOP or α-actin.
(528 KB TIF)

Figure S9 *Atf4*, *Chop*, and *Gadd34* mRNAs Are Unstable Expression of *Atf4, Chop, Gadd34, BiP, Grp94,* and *p58^IPK^* mRNA in the presence or absence of ER stress and actinomycin D was quantitated by real-time RT-PCR as in Figure 5B. The data were normalized against 18S rRNA expression. The expression of each gene at the 0 hour time point for each stressor was set to “1.” Error bars represent means ± SDM from three independent plates.
(291 KB TIF)

Figure S10 *Uggt1* Deletion Results in Up-Regulation of UPR Target Genes (A) The expression of mRNAs for *BiP, p58^IPK^, Chop,* and *Gadd34* in *Uggt1* ^+/+^ and *Uggt1* ^−/−^ MEFs was quantitated by real-time RT-PCR. Error bars represent means ± SDM from RNAs harvested from three separate plates.
(B) E10.5 embryos were harvested from a *Uggt1* ^+/−^; *Uggt1* ^+/−^ cross, and total RNA from whole embryos was prepared for real-time RT-PCR as above. Embryo genotype, determined by PCR from embryonic tissue, was confirmed by *Uggt1* real-time RT-PCR primers, which provide a semi-quantitative assessment of expression of *Uggt1* mRNA.
(308 KB TIF)

Figure S11 Behavior of UPR Target mRNAs and Proteins in Response to a Stress Modeled after Treatment with 25-ng/ml TM Shown are the fluctuations of individual components within the ATF4-CHOP-GADD34 axis and ATF6-BiP axis in response to a modeled stress (shown at upper left), along with a merged plot showing all components together (lower right).
(183 KB TIF)

Figure S12 Behavior of UPR Target mRNAs and Proteins in Response to a Stress Modeled after Treatment with 2.5 nM TG Same as Figure S11, except for 2.5 nM TG instead of 25-ng/ml TM.
(733 KB TIF)

Figure S13 Behavior of UPR Targets as a Consequence of Altering Degradation of CHOP Protein The degradation rate of CHOP protein was adjusted to match that of BiP protein within the 25-ng/ml TM context (i.e., Figure S11), and the effects on each component of the model were determined. Expression of CHOP, GADD34, and BiP proteins from this experiment is also shown in Figure 7D.
(661 KB TIF)

Figure S14 Other Manipulations of Degradation Rates Within the 25-ng/ml TM context, the degradation rates of CHOP and GADD34 mRNAs and proteins were individually adjusted to match those of BiP mRNA or protein. The effects of these manipulations on expression of CHOP and GADD34 protein levels are shown, to allow for comparison with Figure 7C and 7D. Top panel: *Chop* mRNA degradation adjusted to the rate of *BiP* mRNA; middle panel: *Gadd34* mRNA degradation adjusted to the level of *BiP* mRNA; bottom panel: GADD34 protein degradation adjusted to the rate of BiP protein.
(686 KB TIF)

Figure S15 Up-Regulation of *Gadd34* mRNA Is Modest in Adapted Cells Expression of *Gadd34* mRNA in cells treated acutely or chronically with 2.5 nM TG or 25-ng/ml TM was quantitated by real-time RT-PCR from the same RNAs exactly as in Figure 5A.
(592 KB TIF)

Protocol S1 Supplemental Materials, Methods, and Discussion (94 KB DOC)

Table S1 Categorization of ER Morphology Combined from Two Independent EM Experiments (27 KB DOC)

### Accession Numbers

The GenBank (http://www.ncbi.nlm.nih.gov/Genbank) accession numbers for the genes and gene products discussed in this paper are ATF4 (NM_009716), ATF6 (XM_992568), BiP (NM_022310), calnexin (NM_007597), calreticulin (NM_007591), CHOP/GADD153 (NM_007837), eIF2α (NM_026114), GADD34 (NM_008654), GRP94 (NM_011631), IRE1α (NM_023913), p58^IPK^ (U28423), PDI (NM_011032), PERK (NM_010121), Uggt1 (NM_198899), and Xbp1 (NM_013842).

## Abbreviations

ER: endoplasmic reticulum
MEF: mouse embryonic fibroblast
SDM: standard deviation of the mean
TG: thapsigargin
TM: tunicamycin
UPR: unfolded protein response
