# Supplementary material for: Adaptation to ER Stress Is Mediated by Differential Stabilities of Pro-Survival and Pro-Apoptotic mRNAs and Proteins
Source: PLoS Biol. 2006 Nov 7;4(11):e374. doi: 10.1371/journal.pbio.0040374 (PMC1634883; doi:10.1371/journal.pbio.0040374)
Supplement: Supplementary file 1 [file pbio.0040374.sd001.doc]

# Protocol S1 for:

# Adaptation to ER stress is mediated by differential stabilities of pro-survival and pro-apoptotic mRNAs and proteins

# D. Thomas Rutkowski, Stacey M. Arnold, Corey N. Miller, Jun Wu, Jack Li, Kathryn M. Gunnison, Kazutoshi Mori, Amir A. Sadighi Akha, David Raden, and Randal J. Kaufman

#  Supplemental Materials and Methods

*Culture of Factor VII-expressing cells*

CHO cells stably transfected with empty TRex vector or vector containing human coagulation Factor VII (generated and provided by J. J. Hansen, Novo Nordisk, Denmark) were cultured in Ham’s F-12 media supplemented with Glutamax, antibiotics, 10% fetal bovine serum, and 5 μg/ml Vitamin K1. Factor VII expression was induced by addition of tetracycline to the medium at the indicated concentrations.

*Measurement of cytosolic calcium*

Cytosolic Ca^2+^ measurement was performed as previously described[1], with certain modifications. Mouse embryonic fibroblasts (either untreated or treated overnight with 2.5 nM TG) were loaded with 2.5 μM Indo-1 AM (EMD) at 37° for 30 min, washed and then re-suspended in Hanks’ balanced salt solution (HBSS). The Ca^2+^ mobilization assay was conducted at 37° on a FACSVantage flow-cytometer using FACSDiva software (BD Biosciences, San Jose, CA). Baseline levels were collected for 30 s, after which the cells were stimulated with 1 µM TG. The acquired data were analyzed using FlowJo's kinetic platform (TreeStar, Ashland, OR). Each trace displays the mean of the violet : blue ratio as a function of time with smoothing for moving average. The peak increase above baseline was calculated for every sample in each group of fibroblasts, with the data then analyzed using Wilcoxon matched pairs test. Significance was set at p ≤0.05.

*Generation of a mathematical model*

A model describing the expression of components of the ATF4-CHOP-GADD34 and ATF6-BiP signaling axes was made using first order rate constants derived from the experimental data. ER stress/UPR pathways were assumed to be equally induced and proportional to the stress burden, so that *Xbp1* mRNA splicing data (a measure of stress) could be used to approximate levels of ATF4 and ATF6 proteins (i.e., a three-fold increase in Xbp1 splicing was taken as equivalent to a three-fold increase in production of ATF4 or ATF6). The experimental *Xbp1* data (Figs. 2B, 7A and data not shown) were fit to a first order transfer function for a step response. The step response was used in order to fit a single first order equation to the increasing and decreasing trajectories of the stress response. Degradation rate constants were derived from measurements of protein and mRNA half-lives (Figs. 5B, 5D, 5E, and S9; also data not shown). Production rate constants were derived from measurements of steady-state levels of CHOP, GADD34, and BiP protein and mRNA (Figs. 3A, 3C, 5A, and 7B; also data not shown), accounting for the contribution of degradation to the steady state level based on the calculated degradation rate constants. Under this model, ATF4 was directly upstream of CHOP, which was directly upstream of GADD34; while ATF6 was directly upstream of BiP. No interdependence between the two pathways was modeled. The data were derived from both TM and TG experiments, and were used to generate uniform production and degradation rate constants that could be applied to any stress condition, and that could be manipulated as desired. The differential equations were solved in Matlab (The MathWorks, Inc.) using ODE15s. The system was run to steady state for setting initial conditions before inducing the simulated stress.

# *Equations*

## Steady State Conditions, unmanipulated degradation rates (arbitrary units)

Stress = ATF4_pro_ = ATF6_pro_ = %XBP1 splicing (range 1-100)

CHOP_mRNA_ = 100

CHOP_pro_ = 1010

GADD34_mRNA_ = 100

GADD34_pro_ = 1000

BiP_mRNA_ = 1000

BiP_pro_ = 301000

K = 110

tau = 150000

### Rate Constants (sec^-1^)

k_1_ = 6.20E-03	CHOP transcription

k_2_ = 1.00E-03	CHOP translation

k_3_ = 6.1E-06	GADD34 transcription

k_4_ = 0.00126	GADD34 translation

k_5_ = 0.0167	BiP transcription

k_6_ = 0.00166	BiP translation

k_7_ = 6.55E-05	CHOP mRNA degradation

k_8_ = 9.13E-05	CHOP protein degradation

k_9_ = 5.78E-05	GADD34 mRNA degradation

k_10_ = 6.42E-05	GADD34 protein degradation

k_11_ = 2.14E-05	BiP mRNA degradation

k_12_ = 4.19E-06	BiP protein degradation

# Supplemental Discussion

Construction and Utility of a Mathematical Model to Describe the ATF4-CHOP-GADD34 axis of the UPR

Our experimental data demonstrate that ATF4, CHOP, and GADD34 are marked by rapid degradation at both the protein and mRNA levels, in contrast to the protein and mRNA of BiP, GRP94, and p58^IPK^. However, it is not currently possible to selectively experimentally manipulate the degradation rates of these components. Therefore, a mathematical model that can capture the basic production and degradation dynamics of the pathway can be used instead to predict how altered degradation rates might affect protein expression.

The multi-pronged nature of the UPR and the many instances of feedback, both positive and negative, that it utilizes place it beyond the scope of this work to model the entire response in any meaningful way. Instead, we sought to describe the expression of components downstream of both ATF4 and ATF6 utilizing simplifying assumptions that would allow a rudimentary, but nonetheless biologically relevant, model to be constructed. The most significant of these assumptions was that production of ATF4 and ATF6 would occur to a level commensurate with the stress level, and that both proteins would have identical production and degradation rates. While this assumption is almost certainly a dramatic simplification of UPR activation, it allowed us to more specifically dissociate the influence of degradation rate on protein levels because it guarantees that up to the production of *Chop* mRNA or *BiP* mRNA, both pathways behave equivalently. In addition, the assumption is probably reasonable to a first approximation based on several experimental observations. Most notably, we have observed that the levels of ATF4 and the active form of ATF6 seem to follow similar kinetics to splicing of *Xbp1* mRNA, which we take as a measure for the stress burden in the cell. That is, maximal production of ATF4 and ATF6 is seen along a similar time-course as maximal splicing of *Xbp1* (data not shown). In addition, the active form of ATF6α is known to be unstable[2], with a half-life of ~2h, which is similar to our measured half-life of ATF4. Therefore, in our mathematical model, the input “stress” is in reality an estimate of ATF4 or ATF6 levels designed to approximate the level of ER stress.

Another major simplifying assumption was that ATF4 directly and exclusively controls the expression of CHOP and CHOP directly and exclusively controls the expression of GADD34. Likewise, we assumed that BiP expression is controlled directly and exclusively by ATF6. While these dependencies have been established in the published literature, there are almost certainly additional regulatory mechanisms that control expression of these genes. Most notably, it has been suggested that CHOP expression is also regulated by ATF6[3]. Because the full extent of such interactions is not known, we chose to consider only the single and direct controlling interactions outlined above.

Using these assumptions, we incorporated our experimental data into the differential equations described in the Supplemental Methods section.

# Limitations of the Model

As mentioned above, this model does not take into account any of the feedback mechanisms known to act on the UPR, such as GADD34-mediated dephosphorylation of eIF2α or p58^IPK^-mediated suppression of PERK. These and other regulatory steps are undoubtedly important in controlling the output of the UPR, particularly during the adaptation/recovery phase of the response that is the subject of this work. The usefulness of the mathematical model lies more in illustrating the effects that degradation rates alone have in a multistep signaling cascade, providing a glimpse of the extent to which the response can be controlled strictly by the intrinsic properties of its components.

In addition, while the experimental data presented in this paper provide proof-of-principle that cells are capable of surviving fairly constant long-term stresses, the mathematical model is not sophisticated enough to predict in a meaningful way how the UPR will respond to chronic low-level stress (i.e., when adapted cells reach an equilibrium where the UPR is activated to some very low extent). This is largely a consequence of the fact that the sensitivities of our assays for UPR activation are intrinsically limited. Experimentally, we cannot distinguish whether adapted cells reach a state where they do not activate the UPR at all (in such a case, upregulation of BiP would have to be maintained by a different mechanism), or whether UPR activation persists in these cells to a very slight extent. Likewise, where increases in CHOP or GADD34 proteins are undetectable, we cannot exclude the possibility of slight upregulation of these proteins to levels below the sensitivity of immunoblot. Therefore, the relationships between these proteins when the UPR is only slightly activated are not sufficiently well-defined to allow us to incorporate them into a model, although this remains a topic of ongoing investigation.

Finally, while the model seems to approximate our experimental data fairly well, one noticeable discrepancy between the two, that is particularly evident during the TG simulation (Fig. S13), is that the peak inductions of CHOP and GADD34 occur significantly later in the modeled response than in the real experimental response. Specifically, in response to 2.5 nM TG, peak CHOP and GADD34 upregulation occurs in the 4-8 hour range (Figs. 3A, 3C), while the model predicts peak induction of these proteins at approximately 10 and 18 hours, respectively (Fig. S13). The model as constructed assumes constant degradation rates for all components independent of the stress condition. Invoking accelerated degradation of components of the CHOP-GADD34 axis during the adaptive phase of the response (i.e., as the stress burden diminishes) would more closely align the model’s predictions with our data (data not shown). For our calculations of mRNA and protein half-lives, cells were first pretreated with TM or TG to induce UPR activation. Thus, the measured half-lives of the various components are most relevant to the adaptive phase of the response, and it is possible that degradation during the first phase of UPR activation (i.e., during an increasing stress burden) is slower; other studies have suggested that CHOP stability can indeed be regulated[4-7]. Identifying the factors responsible for mediating degradation of CHOP and GADD34 should shed further light on how cells adapt to stress and escape apoptosis.

# Supplemental Notes

1. In Figure 1A, we observed that, in response to varying concentrations of TG treatment, BiP protein levels were actually lower at higher doses of TG (e.g., 30-100 nM) than at lower doses. This phenomenon was observed independent of the cell lysis method used, making it unlikely to be an artifact of lysis. More likely, it reflects the robust inhibition of translation seen for higher concentrations of TG (see Fig. 2C and S5), not simply by virtue of suppression of BiP synthesis alone, but instead, or in addition, by suppression of ATF6 synthesis. It has been suggested that the active form of ATF6 is derived from nascent ATF6 synthesized during the stress, rather than from preexisting pools of this sensing molecule[8,9]. Our data support this observation, in that we also detect less cleavage of ATF6α in response to 100 nM TG than in response to 2.5 nM TG (see Fig. 2A, comparing the relative abundance of the cleaved p50 form and the uncleaved p90 form). Therefore, it seems likely that treatment with high concentrations of TG suppresses production of the active form of ATF6α.

2. It could be argued that cells exposed to low doses of a pharmacological inducer of ER stress might lose their pharmacological sensitivity to the substance, and that the attenuated induction of the UPR seen in adapted cells might be a consequence of such pharmacological tolerance, rather than true adaptation by an increase in the protein folding and processing capacity of the cell[10]. To explore this possibility for TM, we compared the inhibition of glycosylation in cells adapted to TM for >4 days to inhibition in newly exposed cells (a minimum of 4 days of adaptation was chosen because this was a time-frame that, in other experiments, was needed for cells to reach an apparent steady state with respect to their attenuated response to TM; see for example Fig. 3B). This analysis showed that, immediately after exposure to TM, naïve cells show very little inhibition of glycosylation, while inhibition in adapted cells is substantial (Fig. S3). The apparent robust inhibition in adapted cells is probably a reflection of the cumulative effects of the newly added TM and TM remaining from the previous day’s treatment. After approximately 24 hours of treatment, inhibition of glycosylation in both naïve and adapted cells is low, and thus we cannot definitively exclude that adapted cells might perhaps metabolize TM somewhat more rapidly than naïve cells. However, the data do not support any gross discrepancy between naïve and adapted cells in terms of their sensitivity to the pharmacological effects of TM.

Similarly, we compared the Ca^2+^ response of untreated MEFs with that of MEFs exposed to 2.5 nM thapsigargin overnight. In this case, only one day of adaptation was used because, in other experiments, we found that 24 hours of treatment with TG was all that was necessary for adapted cells to display resistance to UPR induction by further TG treatment. Untreated and thapsigargin-treated cells were loaded with Indo-1, and exposed to 1 μM TG to inhibit the ER Ca^2+^-ATPase and induce an increase in cytosolic Ca^2+^ levels. The dose of 1 μM is typical for this purpose, and has previously been used to investigate the role of Bax inhibitor-1 (BI-1) in regulating ER handling of the Ca^2+^ response[11], wherein a clear difference between the calcium fluxes of wild-type and knockout cells was observed. In our experiments, exposure to 1 μM thapsigargin induced a similar increase in cytosolic Ca^2+^ in untreated MEFs and in MEFs exposed to 2.5 nM thapsigargin overnight (p=0.5). The assay is not sensitive enough to detect calcium flux in response to 2.5 nM TG (data not shown), so we cannot rule out small differences in pharmacological sensitivity to TG, but as with TM, the loss of pharmacological sensitivity seems to be an unlikely explanation for adaptation to ER stress.

3. The formulae used to describe “stress” in the mathematical model, and thus the levels of ATF4 and ATF6, result in a curve that represents only a first approximation of the stress experienced by the cell, incorporating real data points for *Xbp1* mRNA splicing. The true trajectory of the stress response in an adapting or adapted cell is not known. However, we found, by manipulating the trajectory of the stress curves, that these changes had no significant effect on the responses of the downstream components to the stress, nor on the changes in the responses caused by manipulation of degradation rates.

4. Slowing the degradation rates of components, such as CHOP protein, in the mathematical model results in an expression peak that is less responsive to the attenuation of stress, but also reaches a lower maximum induction. Alterations in degradation rate were first used to adjust the steady-state (i.e., non-stressed) conditions upon which the model was based, meaning that these components are then induced under stressed conditions relative to a different initial state. However, because the production and degradation dynamics of CHOP and GADD34 in unstressed conditions are not known, and because their expression, at least at the protein level, is probably very near zero, it is not clear whether this effect (reduction in peak height) is physiologically relevant or not. Thus, the kinetic behavior of the response curves (their responsiveness to stress) is likely of more relevance than their amplitudes.

5. The direct detection of activation of UPR stress sensors in *Uggt1*-/- cells (Fig. 6) seemingly raises the question of why these events can be detected in these cells but not readily in pharmacologically adapted cells (Fig. 4). We have observed that the activation of UPR sensors, assessed by both direct and indirect measures, becomes more prominent as the passage number of the cells increases (all experiments shown were carried out on passage-matched wild-type and *Uggt1*-/- cells cultured between 10-15 passages). Earlier passage cells still demonstrate evidence for UPR activation, but it appears to be less robust. It seems likely that the protein folding problems caused by *Uggt1* deletion are at least partially cumulative in inducing ER stress.

6. Several recent papers have highlighted potential contributions of the IRE1-XBP1 pathway of the UPR in adaptation[12,13] and apoptosis[14]. In our experimental system, we find IRE1 to be dispensable for adaptation, as *Ire1*-/- cells are capable of proliferation in low concentrations of TM, and of upregulation of BiP and GRP94 expression under such conditions (Fig. S6). However, it seems likely that the signaling cascades emanating from IRE1 activation would also be structured to favor adaptation in cell types where IRE1 function is more critical.

References

1. Sadighi Akha AA, Berger SB, Miller RA (2006) Enhancement of CD8 T-cell function through modifying surface glycoproteins in young and old mice. Immunology [Epub ahead of print] PMID: 16805788.

2. Haze K, Yoshida H, Yanagi H, Yura T, Mori K (1999) Mammalian transcription factor ATF6 is synthesized as a transmembrane protein and activated by proteolysis in response to endoplasmic reticulum stress. Mol Biol Cell 10: 3787-3799.

3. Ma Y, Brewer JW, Diehl JA, Hendershot LM (2002) Two distinct stress signaling pathways converge upon the CHOP promoter during the mammalian unfolded protein response. J Mol Biol 318: 1351-1365.

4. Bartlett JD, Luethy JD, Carlson SG, Sollott SJ, Holbrook NJ (1992) Calcium ionophore A23187 induces expression of the growth arrest and DNA damage inducible CCAAT/enhancer-binding protein (C/EBP)-related gene, gadd153. Ca2+ increases transcriptional activity and mRNA stability. J Biol Chem 267: 20465-20470.

5. Hattori T, Ohoko N, Inoue Y, Hayashi H, Onozaki K (2003) C/EBP family transcription factors are degraded by the proteasome but stabilized by forming dimer. Oncogene 22: 1273-1280.

6. Jackman J, Alamo Jr. I, Fornace Jr. AJ (1994) Genotoxic stress confers preferential and coordinate messenger RNA stability on the five gadd genes. Cancer Res 54: 5656-5662.

7. Ubeda M, Schmitt-Ney M, Ferrer J, Habener JF (1999) CHOP/GADD153 and methionyl-tRNA synthetase (MetRS) genes overlap in a conserved region that controls mRNA stability. Biochem Biophys Res Commun 262: 31-38.

8. Hong M, Luo S, Baumeister P, Huang JM, Gogia RK, et al. (2004) Underglycosylation of ATF6 as a novel sensing mechanism for activation of the unfolded protein response. J Biol Chem 279: 11354-11363.

9. Hong M, Li M, Mao C, Lee AS (2004) Endoplasmic Reticulum Stress Triggers an Acute Proteasome-Dependent Degradation of ATF6. Journal of Cell Biochem 92: 723-732.

10. Rine J, Hansen W, Hardeman E, Davis RW (1983) Targeted selection of recombinant clones through gene dosage effects. Proc Natl Acad Sci U S A 80: 6750-6754.

11. Chae HJ, Kim HR, Xu C, Bailly-Maitre B, Krajewska M, et al. (2004) BI-1 regulates an apoptosis pathway linked to endoplasmic reticulum stress. Mol Cell 15: 355-366.

12. Tirosh B, Iwakoshi NN, Glimcher LH, Ploegh HL (2006) Rapid turnover of unspliced Xbp-1 as a factor that modulates the unfolded protein response. J Biol Chem 281: 5852-5860.

13. Yoshida H, Oku M, Suzuki M, Mori K (2006) pXBP1(U) encoded in XBP1 pre-mRNA negatively regulates unfolded protein response activator pXBP1(S) in mammalian ER stress response. J Cell Biol 172: 565-575.

14. Hetz C, Bernasconi P, Fisher J, Lee AH, Bassik MC, et al. (2006) Proapoptotic BAX and BAK modulate the unfolded protein response by a direct interaction with IRE1alpha. Science 312: 572-576.

```
% M-file = Mammalian UPR
% Written by David Raden  draden at udel dot edu
% Department of Chemical Engineering
% University of Delware
% Set 7/14/2007


% Setting for Normal response to TG25 using Transfer function to simulate stress


%=======Time of Run in seconds 3600 sec = 1hr====================================


T0= 0; 			% starting time for stress step (k1 = 1)

Ts = 3600*4;	% end time for stress step (k1 = 1) and start time for unstressed step (k1 = 0)

Tf = 3600*48; 	% end time for unstressed step (k1 = 0)


%=======Steady-state Conditions from UPRsteady===================================


ATF4 = 1; 			% XBP splicing=ATF4=ATF6

chopm = 94.656;		% CHOP mRNA

chopP = 1036.78;	% CHOP Protein

gaddm = 109.42;		% GADD34 mRNA = ToCs mRNA

gaddP = 2147.4;		% GADD34 Protein = ToCs Protein

BiPm = 780.37;		% BiP mRNa

BiPP = 309170;		% BiP Protein

%=======rate constants==========================================================


k2 = 6.20E-03   ;	% CHOP transcription

k3 = 1.00E-03	;	% ChOP translation

k4 = 6.1E-06	;	% GADD34 transcripion

k5 = 0.00126	;	% GADD34 translation

k7 = 6.4E-05	;	% ATF4/6 degrad

k8 = 6.55E-05	;	% CHOP mRNA degrad

k9 = 4.19E-06	;	% CHOP protein degrad   9.13E-05

k10 = 5.78E-05 	;	% GADD34 mRNA degrad

k11 = 6.42E-05	;	% GADD34 protein degrad

k12 = 0.0167	;	% BiP transcription

k13 = 0.00166	;	% BiP translation

k14 = 2.14E-05	;	% BiP mRNA degrad

k15 = 4.19E-06	;	% BiP protien degrad

K = 110;

tau = 111000;


%========Calling the solver for start of Stressed============================


k1 = 1; % Stress inducing portion of step function

[T1,X1] = ode15s(@upr, [T0 Ts], [ATF4 chopm chopP gaddm gaddP BiPm BiPP],[],k1,k2,k3,k4,k5,k7,k8,k9,k10,k11,k12,k13,k14,k15,K,tau);

%========Calling the solver for start of Unstressed==========================


k1  = 0; % Unstressed portion of step function

[T2,X2] = ode15s(@upr, [Ts Tf], [X1(end,:)],[],k1,k2,k3,k4,k5,k7,k8,k9,k10,k11,k12,k13,k14,k15,K,tau);


%=======Combining Data from Stessed and Unstressed solutions================


 T = [T1;T2];

 X = [X1;X2];


save uprS2 X -ascii -tabs

save uprT2 T -ascii -tabs

%=======Solver for Mammalian UPR==========================================

function sys = upr(t,x,k1,k2,k3,k4,k5,k7,k8,k9,k10,k11,k12,k13,k14,k15,K,tau)


%======= equations==========================================
sys(1,1) =   ((K*k1 - x(1))/(tau));	% Stress Response: Transfer function of step response optimized to XBP1 data
sys(2,1) = k2*x(1) - k8*x(2);		% CHOP mRNA
sys(3,1) = k3*x(2) - k9*x(3);		% CHOP Protein
sys(4,1) = k4*x(3) - k10*x(4);		% GADD34 mRNA = ToCs mRNA
sys(5,1) = k5*x(4) - k11*x(5);		% GADD34 Protein = ToCs Protein
sys(6,1) =  k12*x(1) - k14*x(6);	% BiP mRNa
sys(7,1) =  k13*x(6)- k15*x(7);		% BiP Protein
```
