# Supplementary material for: Adaptation to ER Stress Is Mediated by Differential Stabilities of Pro-Survival and Pro-Apoptotic mRNAs and Proteins
Source: PLoS Biol. 2006 Nov 7;4(11):e374. doi: 10.1371/journal.pbio.0040374 (PMC1634883; doi:10.1371/journal.pbio.0040374)
Supplement: Supplementary file 2 [file pbio.0040374.st001.doc]

Table S1: Categorization of ER morphology combined from two independent EM experiments.

| Stress | Normal | Dilated | Vesiculated | Fragmented | Miscellaneous Abnormal | Ambiguous | Total |
| --- | --- | --- | --- | --- | --- | --- | --- |
| NT | 12 |  |  |  |  | 1 | 13 |
| Hi TM o/n |  | 10 | 2 |  |  | 2 | 14 |
| 25 ng/ml TM o/n | 1 | 6 | 1 | 2 |  | 2 | 12 |
| 25 ng/ml TM chronic | 11 | 2 | 1 |  |  | 1 | 15 |
| Hi TG o/n | 1 | 1 | 3 | 7 | 1 |  | 13 |
| 2.5 nM TG o/n | 10 |  | 1 | 1 | 1 | 1 | 14 |
| 2.5 nM TG chronic | 9 | 1 |  | 1 | 1 |  | 12 |

“Fragmented” was defined as absence of structurally intact ER evident despite maintenance of nuclear and mitochondrial structure. “Ambiguous” was defined as images that could not be reliably placed into any category. Hi TM = 1 μg/ml or 2 μg/ml TM. Hi TG = 100 or 250 nM TG. Chronic ≥ 6 days treatment.
